# Design of Organic Cathode Material Based on Quinone and Pyrazine Motifs for Rechargeable Lithium and Zinc Batteries

**DOI:** 10.1021/acsami.3c16038

**Published:** 2024-03-21

**Authors:** Svit Menart, Olivera Lužanin, Klemen Pirnat, David Pahovnik, Jože Moškon, Robert Dominko

**Affiliations:** † National Institute of Chemistry, Hajdrihova 19, 1001 Ljubljana, Slovenia; ‡ Faculty of Chemistry and Chemical Technology, University of Ljubljana, Večna pot 113, 1000 Ljubljana, Slovenia; § ALISTORE-European Research Institute, 33 rue Saint-Leu, 80039 Amiens, France

**Keywords:** organic cathode, pyrazine, quinone, catechol, Li−organic battery, Zn−organic battery, aqueous electrolyte

## Abstract

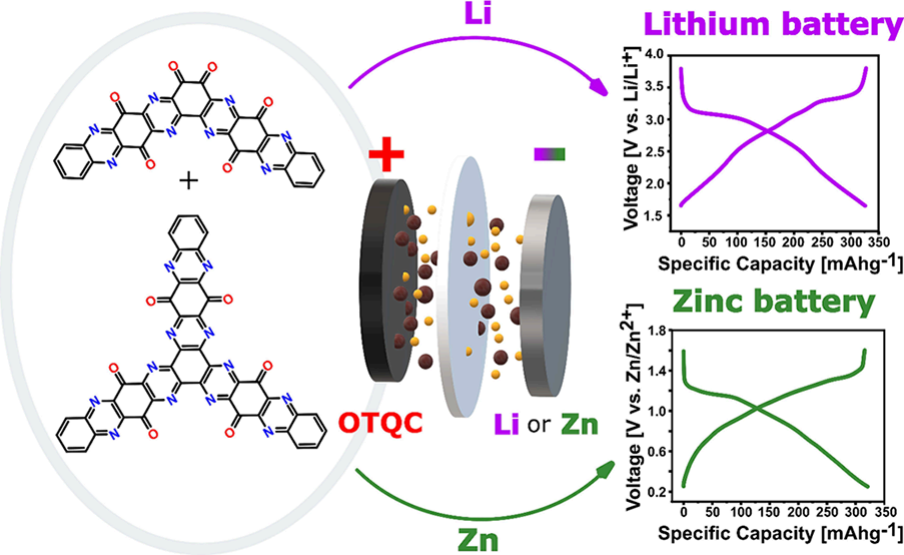

Despite the rapid expansion of the organic cathode materials field, we still face a shortage of materials obtained through simple synthesis that have stable cycling and high energy density. Herein, we report a two-step synthesis of a small organic molecule from commercially available precursors that can be used as a cathode material. Oxidized tetraquinoxalinecatechol (OTQC) was derived from tetraquinoxalinecatechol (TQC) by the introduction of additional quinone redox-active centers into the structure. The modification increased the voltage and capacity of the material. The OTQC delivers a high specific capacity of 327 mAh g^–1^ with an average voltage of 2.63 V vs Li/Li^+^ in the Li-ion battery. That corresponds to an energy density of 860 Wh kg^–1^ on the OTQC material level. Furthermore, the material demonstrated excellent cycling stability, having a capacity retention of 82% after 400 cycles. Similarly, the OTQC demonstrates increased average voltage and specific capacity in comparison with TQC in aqueous Zn–organic battery, reaching the specific capacity of 326 mAh g^–1^ with an average voltage of 0.86 V vs Zn/Zn^2+^. Apart from good electrochemical performance, this work provides an additional in-depth analysis of the redox mechanism and degradation mechanism related to capacity fading.

## 1 Introduction

Since the commercialization of Li-ion batteries by the Sony Corporation in 1991, the cathode material has proven to be the bottleneck for their specific capacities. Because of the rapidly expanding utilization of energy from renewable sources with an intermittent nature, there is an increasing demand for efficient energy storage. The scarcity of transition metal elements such as cobalt and nickel is driving research for a new generation of sustainable and low-cost batteries.

In recent years a more sustainable alternative to the presently used materials has emerged in the form of organic cathode materials, which can be made from readily available raw materials with a lower carbon footprint.^1^ Additionally, organic materials can swell, which can significantly improve ionic conductivity,^2−4^ and can overcome slow solid-state diffusion limitations of inorganic materials enabling their use in multivalent batteries such as aluminum,^5,6^ magnesium,^7,8^ and zinc.^9,10^ Compatibility of the zinc metal with aqueous electrolytes made zinc-ion batteries one of the most promising contenders for large-scale energy storage applications. Apart from inherent safety due to lower toxicity and nonflammability of aqueous electrolytes, the use of zinc metal also offers cost benefits due to higher world resources (approximately 20 more abundant than Li) and the stability of the Zn-battery components in an ambient atmosphere, which simplifies the production process.^11−13^

Despite many reports on new organic cathode materials, there is still a lack of materials obtained through facile synthesis with stable cycling and high energy density. To date, various categories of redox-active organic materials have been studied, including imine compounds,^14^ stable organic radicals,^15^ conductive polymers,^16^ nitro aromatics,^17^*N*,*N*′-substituted phenazines,^18^ and most extensively studied conjugated carbonyl compounds.^19^ Introducing additional redox-active motifs into organic materials structure increases the theoretical capacity of the system. A recent study showed that a simple substitution of the benzene unit with pyrazine in the anthraquinone molecule simultaneously increased the voltage and the theoretical capacity of the system.^20^ Several recent reports employed the findings and constructed small organic cathode materials with multiple pyrazine and quinone units displaying excellent performance in Li–organic^21−25^ and Zn–organic batteries.^9,26−30^ Although small organic cathode materials exhibit high specific capacities, most are plagued by fast capacity fading due to dissolution in the electrolyte. Several strategies have been used to address the problem, such as immobilization of the active material on the solid support,^31^ use of concentrated electrolytes,^32^ use of selective separators,^33^ and inclusion of the redox-active unit into a less soluble polymer.^34^ Implementation of the presented approaches comes with a drawback of decreased energy density of the system due to the addition of non-redox-active components.

In our recent report,^35^ we explored a facile synthesis strategy for the simultaneous inclusion of catechol and pyrazine units into organic cathode materials. Extending the molecular structure with additional quinoxaline units enabled newly synthesized tetraquinoxalinecathechol (TQC) to deliver one of the best cycling stabilities of small organic materials in a Li–organic battery, reaching a capacity retention of 82% after 300 cycles at a current of 50 mA g^–1^. In the Zn–organic battery with the aqueous electrolyte, TQC showed higher capacity in comparison to the Li–organic battery but worse cycling stability.

Herein, we report a simple derivation of TQC by adding additional quinone units into the structure of the molecule through facile oxidation with potassium dichromate. The synthesized TQC in a fully oxidized form (OTQC) exhibited improved electrochemical performance in the Li–organic battery, delivering reversible specific capacities of 327 mAh g^–1^ at 50 mA g^–1^ with an average voltage of 2.63 V versus lithium metal. The cycling stability of the half-cell with OTQC-active material showed a high capacity retention of 82% after 400 cycles at a current density of 50 mA g^–1^, which is highly improved compared to most reported small organic cathode materials (Table S2). OTQC was also evaluated in a Zn–organic battery, where it showed increased voltage and specific capacity in comparison with TQC. The redox mechanisms and capacity fading were thoroughly investigated using multiple spectroscopic and electrochemical methods.

## 2 Results and Discussion

**Figure 1:**
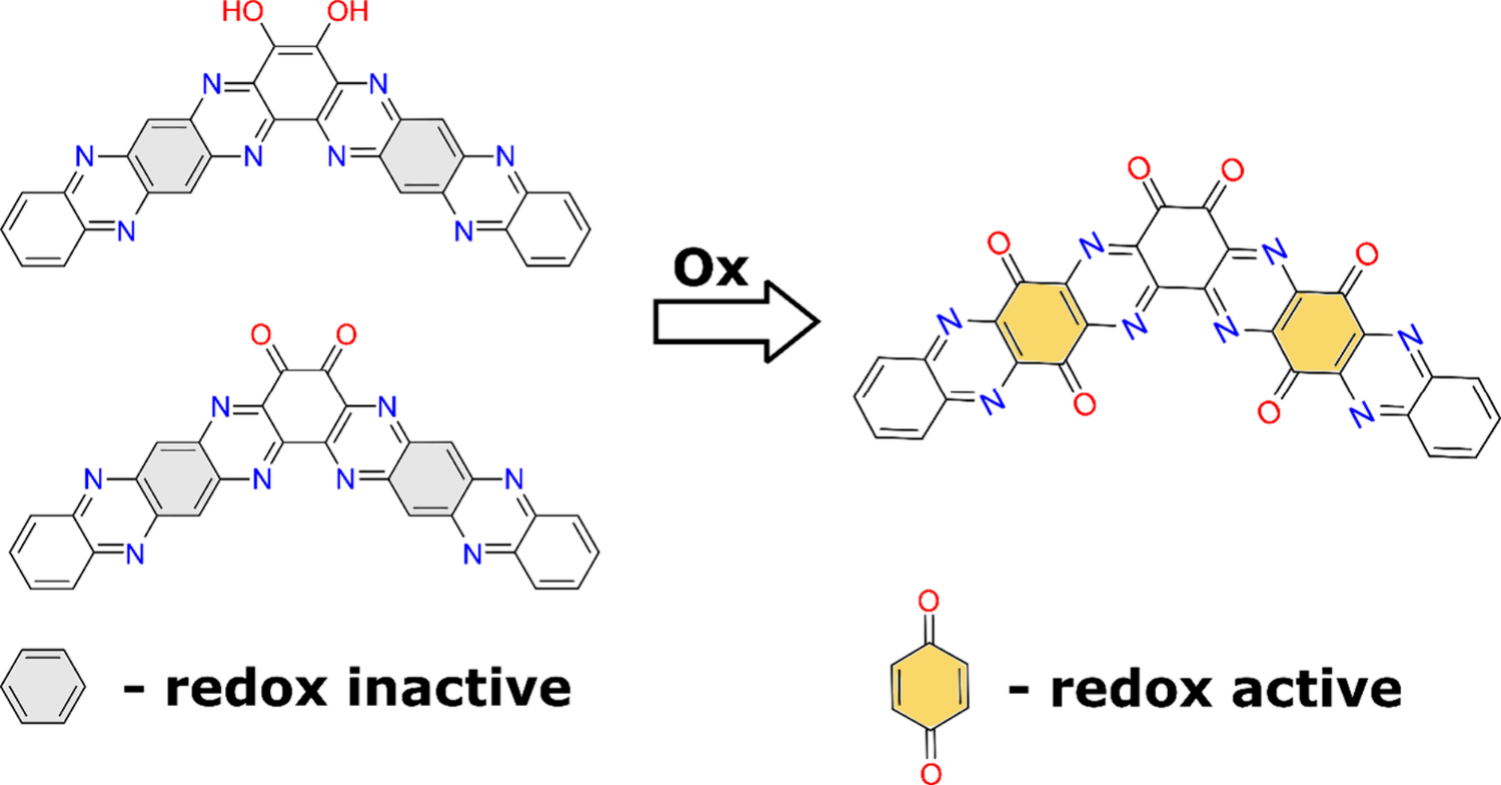
Design optimization strategy using oxidation to transform redox-inactive benzene rings into redox-active quinones enabling the increase of the theoretical capacity.

**Figure 2:**
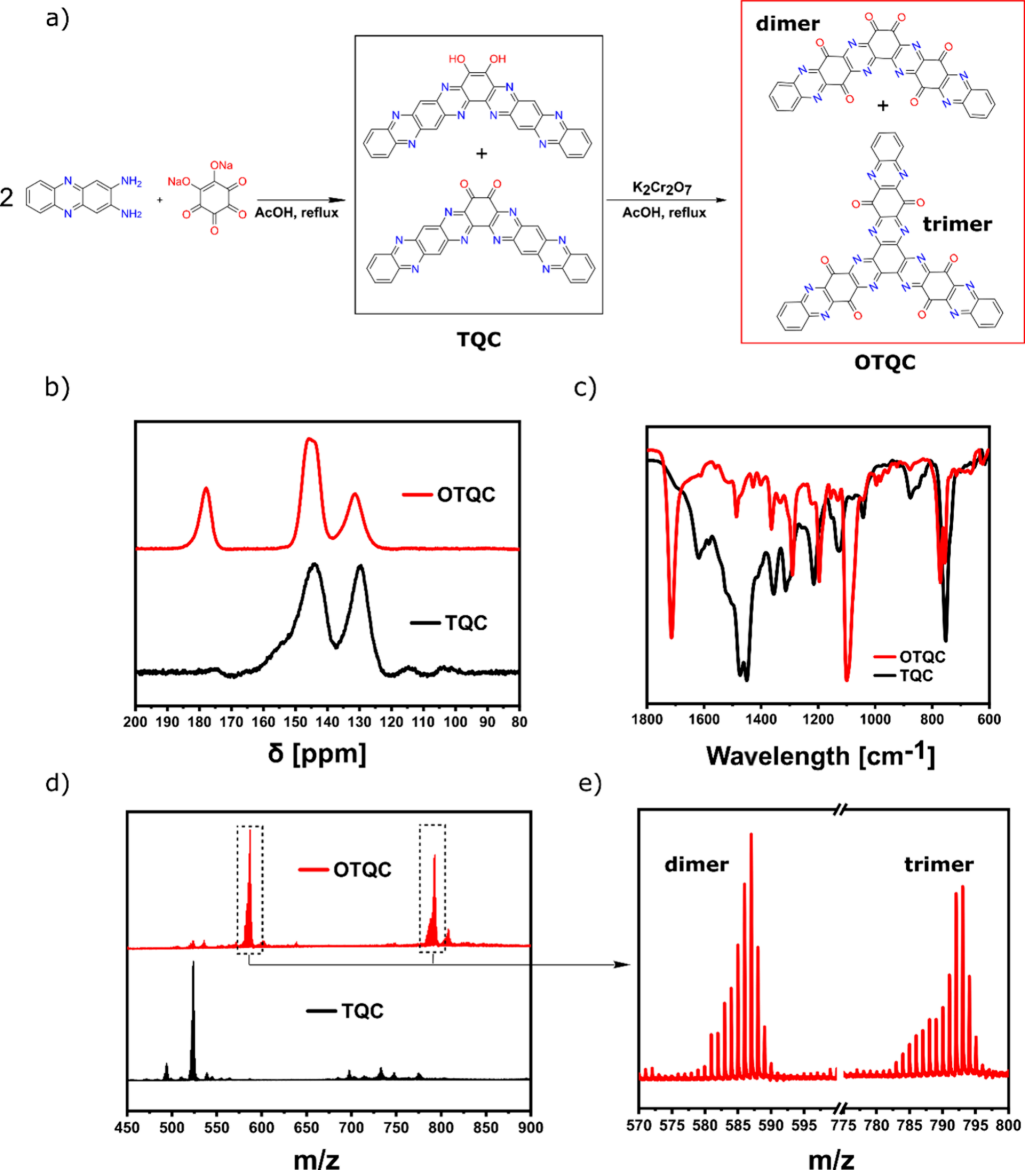
(a) Synthesis of OTQC. (b) Comparison of ^13^C MAS NMR spectra between TQC (black) and OTQC (red). (c) Comparison between FT-IR spectra of TQC (black) and OTQC (red). (d) Comparison of MALDI-TOF MS spectra between TQC (black) and OTQC (red). (e) Zoomed-in MALDI-TOF MS spectrum of OTQC.

Intending to improve the TQC material,^35^ we introduced the additional redox-active motifs into the structure. That was done by replacement of the redox-inactive benzene units with the redox-active quinone units (Figure 1). A similar transformation has already been applied in organic cathode materials, where the oxidation of the benzene unit was achieved by refluxing the precursor with potassium dichromate (K_2_Cr_2_O_7_) in diluted sulfuric acid.^25^ Employing the same conditions for the oxidation of TQC led to only a trace amount of insoluble product, presumably because of too harsh conditions. Using less harsh conditions by replacing the solvent with glacial acetic acid solved the problem, and we obtained OTQC (Figure 2a). Similar to the TQC, OTQC’s low solubility in the commonly used NMR solvents prevented the characterization with liquid NMR. Successful oxidation of the material was therefore confirmed with the use of multiple other techniques. A comparison of the ^13^C MAS NMR spectra between TQC and OTQC shows the emergence of another peak at 177.9 ppm attributed to the carbonyl unit (C=O) in the newly formed quinone structures (Figure 2b, red). Furthermore, the introduction of additional carbonyl units was confirmed by FT-IR with the appearance of a strong peak at 1714 cm^–1^ attributed to the (C=O) stretching vibrations (Figure 2c). Successful oxidation of TQC was additionally confirmed with MALDI-TOF MS spectra, which revealed the disappearance of peaks associated with TQC (Figure 2d, black) and the emergence of peaks at higher *m*/*z* attributed to the oxidized products (Figure 2d, red). Although we expected the formation of pure dimer OTQC (Figure 2a), MALDI-TOF MS spectra revealed two major sets of peaks: one at around 586 Da attributed to the formation of the dimer OTQC and another at around 793 Da attributed to the formation of the trimer OTQC with a hexaazatriphenylene core (Figure 2e). A possible explanation for the formation of the trimer OTQC was derived from the low yield of the synthesis (42%), suggesting a partial hydrolysis of TQC during the oxidation. According to the literature,^36^ we presume that there is a dynamic reversible reaction in the inner pyrazine units of TQC or its oxidation products, which can be hydrolyzed to aromatic diamines and reformed. Formation of the hexaazatriphenylene core is achieved through the reaction of the aromatic diamine and the free *o*-benzoquinone unit in TQC. We need to emphasize that both products are electrochemically active; however, the trimer has a lower specific capacity.

**Figure 3:**
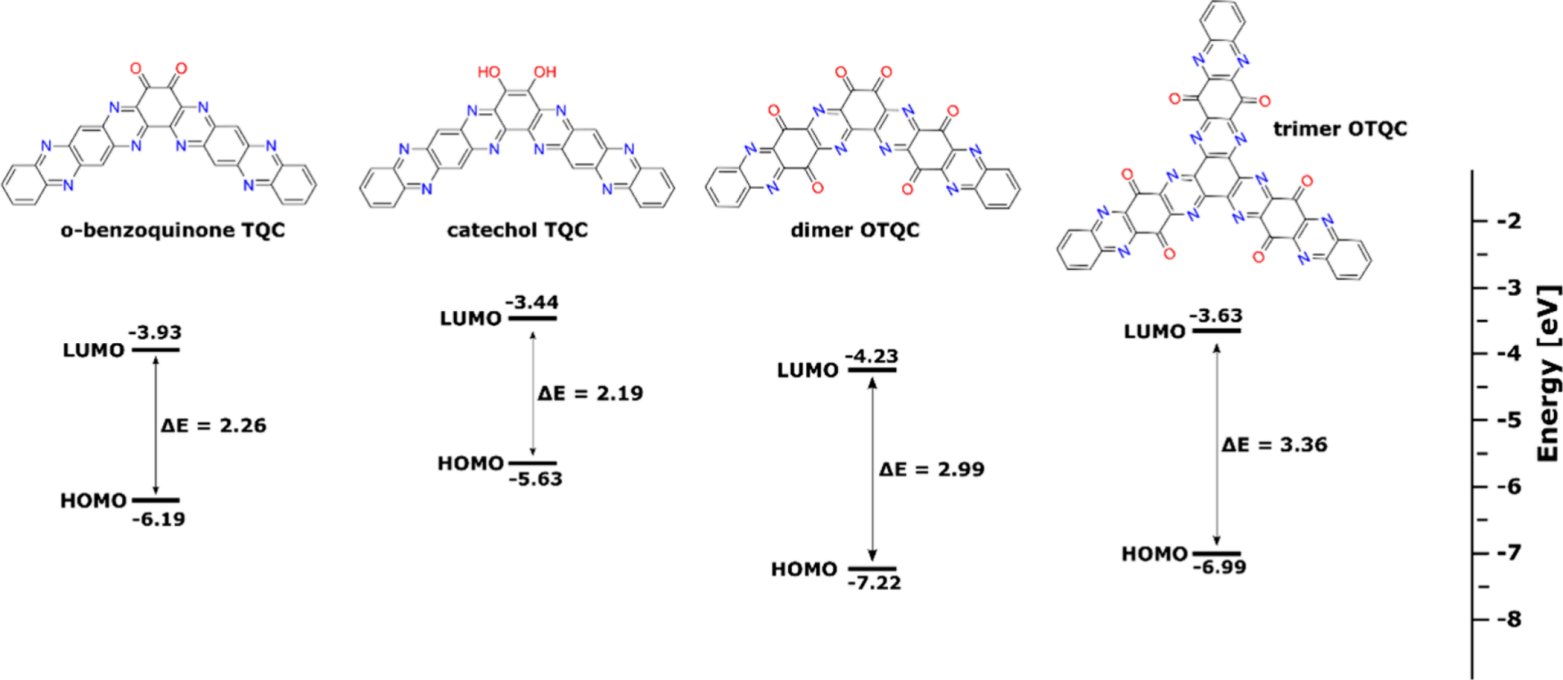
HOMO/LUMO energy levels of *o*-benzoquinone TQC, catechol TQC, dimer OTQC, and trimer OTQC.

HOMO/LUMO energy levels for compounds studied in this work (Figure 3) were calculated using density functional theory (DFT). The comparison between TQC and OTQC revealed that TQC exhibits a lower energy gap (*E*_g_) between HOMO and LUMO orbitals, which suggests that oxidized products possess lower intrinsic electronic conductivity. The calculations showed a big variability between energy levels of the LUMO orbitals connected to the electronic affinity of the molecules ranging from the lowest value of −4.23 eV in the dimer OTQC to the highest −3.44 eV in catechol TQC. Lower LUMO energy, lower energy gap, and higher amount of redox-active centers within the dimer OTQC in comparison with the trimer OTQC suggest the improved electrochemical properties of the dimer OTQC.

**Figure 4:**
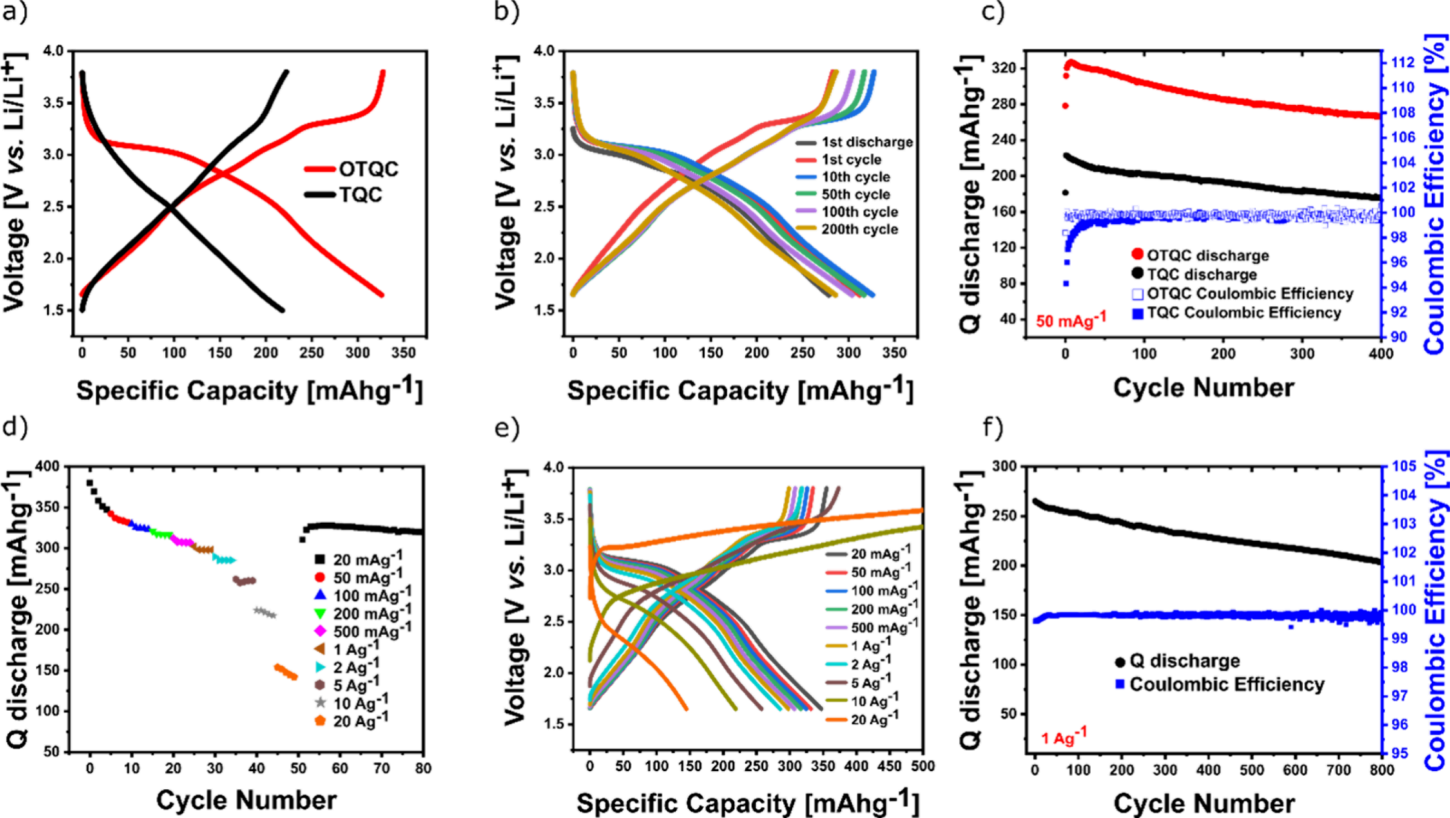
(a) Comparison of the galvanostatic charge/discharge curves between TQC (black, 2nd cycle) and OTQC (red, 2nd cycle) at 50 mAg^–1^. (b) Galvanostatic charge/discharge curves of OTQC at 50 mA g^–1^. (c) Cycling stability of OTQC (red) and TQC (black) at 50 mA g^–1^. (d, e) Rate performance of OTQC. (f) Cycling stability of OTQC at 1 A g^–1^.

The galvanostatic experiments were performed in a Swagelok cell using an organic cathode and metallic lithium partitioned by the separator soaked with 1 M lithium bis(trifluoromethanesulfonyl)imide (LiTFSI) in 1:1 (v/v) 1,3-dioxolane (DOL) and 1,2-dimethoxyethane (DME) as an electrolyte in the voltage ranges 1.5–3.8 and 1.65–3.8 V for TQC and OTQC, respectively. Using a wider voltage window of 1.5–3.8 V for OTQC resulted in higher specific capacity but lower cycling stability (Figure S1). The comparison of galvanostatic charge–discharge curves between TQC and OTQC showed considerable differences (Figure 4a). Charge/discharge curves of the TQC exhibited a single-sloping curve. On the other hand, the OTQC exhibited a distinct discharge plateau at around 3.1 V, which gradually progressed into a sloping curve. The galvanostatic measurements revealed a clear benefit of the oxidation step, which increased the voltage and the capacity of the system. OTQC reached the highest capacity of 327 mAh g^–1^ (comprising a 22 mAh g^–1^ capacitive contribution of Printex XE2 carbon black, Figure S2) at 50 mA g^–1^ with an average voltage of 2.63 V, corresponding to one of the highest reported energy densities of 860 Wh kg^–1^ for organic materials. In comparison, TQC reached the highest capacity of 223 mAh g^–1^ with an average voltage of 2.42 V. The oxidation step did not compromise the cycling stability, which demonstrated capacity retention of 82% and 79% after 400 cycles at 50 mA g^–1^ for the OTQC and TQC, respectively (cycling time of 194 days vs 129 days) (Figure 4b,c). Despite possessing very high theoretical capacities based on the reduction of all the pyrazine and the quinone redox-active centers (dimer OTQC, 651 mAh g^–1^ (6 e^–^ exchange for quinones + 8 e^–^ exchange for pyrazines), and trimer OTQC, 619 mAh g^–1^ (8 e^–^ exchange for quinones + 12 e^–^ exchange for pyrazines), the sloping voltage profile prevents the full capacity utilization due to the limited stability window of the DOL and DME electrolyte (Figure S3a). A similar problem has been reported in the literature, where it was solved with the use of different binder and electrolyte systems.^21^ To test the performance in wider voltage window, additional experiments were conducted in 1 M lithium hexafluorophosphate (LiPF_6_) in 1:1 (v/v) ethylene carbonate (EC) and dimethyl carbonate (DMC) electrolyte (LP30), which enables cycling to lower voltage of 1.2 V (Figure S3b). Utilizing a wider voltage window between 1.2 and 3.8 V in the LP30 electrolyte substantially increased the capacity, delivering 507 mAh g^–1^ in the first discharge (comprising 34 mAh g^–1^ capacitive contribution of Printex XE2 carbon black, Figure S5) with an average voltage of 2.29 V. This corresponds to 73% and 76% of the theoretical capacity utilization based on OTQC dimer and OTQC trimer, respectively. On the other hand, cycling to lower voltages decreased the cycling stability, reaching 23% after 60 cycles at 50 mA g^–1^ (Figure S4). OTQC exhibited a great rate performance; at the highest rate of 20 A g^–1^ the system reached a capacity of 148 mAh g^–1^ (39% of the maximum capacity) (Figure 4d). The system exhibited high overcharging at currents greater than 5 A g^–1^, which indicated irreversible reactions during the oxidation process (Figure 4e). Despite excellent long-term stability at lower rates, OTQC showed moderate cycling stability at a high current of 1 A g^–1^, delivering a capacity retention of 77% after 800 cycles (cycling time of 17 days) (Figure 4f).

**Figure 5:**
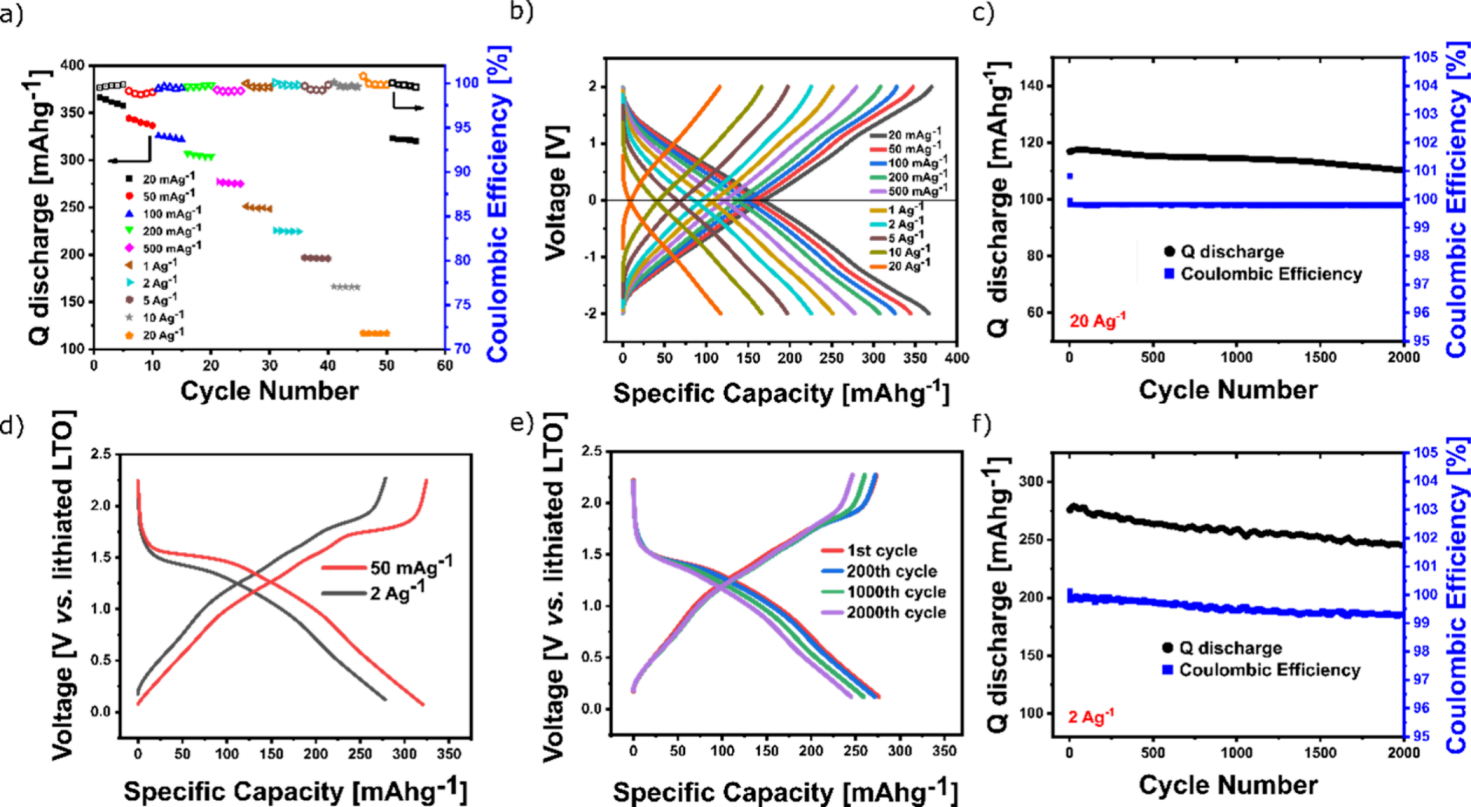
(a, b) Rate performance test of OTQC in a symmetric cell. (c) Cycling stability of OTQC in a symmetric cell at 20 A g^–1^. (d) Galvanostatic charge/discharge curves of the OTQC-LTO battery at 50 mA g^–1^ (red) and 2 A g^–1^ (black). (e) Galvanostatic charge/discharge curves of the OTQC-LTO battery at 2 A g^–1^. (f) Cycling stability of the OTQC-LTO battery at 2 A g^–1^.

Two approaches were used to test the hypothesis that the cycling stability issues and overcharging observed at higher current densities were a consequence of the use of a lithium metal counter electrode. Our previous work showed that the use of organic symmetric cells can eliminate the problems associated with the metal anodes and reveal the true limits of the organic materials.^37^ Transferring this approach to the OTQC proved to be challenging due to the high potential and asymmetry of the voltage curves. Based on the difference between the average voltage (2.63 V) and the lower potential limit of the OTQC-Li battery (1.65 V), the voltage window of (−2 to +2 V) was chosen as the best compromise in terms of the electrolyte stability and the appropriate representation of cycling in the OTQC-Li battery. The OTQC symmetric cell was constructed from an OTQC electrode discharged to 1.65 V and a pristine OTQC electrode partitioned by a Celgard separator wetted with an additional electrolyte. The rate performance test of the OTQC symmetric cell showed values of discharge capacities comparable to those of the OTQC-Li battery but did not show overcharging at higher current densities (Figure 5a,b).

In contrast to the cell with a lithium metal anode, the symmetric cell showed excellent cycling stability even at the high current density of 20 A g^–1^, reaching a capacity retention of 94% after 2000 cycles with Coulombic efficiency close to 100% (Figure 5c). To further confirm the hypothesis, we substituted the lithium metal anode with an inorganic anode material, lithiated lithium titanate (LTO), which exhibits a stable voltage plateau around 1.55 V vs Li/Li^+^ (Figure S6).^38^ The OTQC-LTO battery showed voltage curves similar to those of the OTQC-Li battery transposed by approximately 1.55 V (Figure 5d). Similarly to the OTQC symmetric cell, no overcharging was observed at a high current density of 2 A g^–1^, enabling stable cycling reaching a capacity retention of 88% after 2000 cycles with Coulombic efficiency close to 100% (Figure 5e,f). The experiments proved that the charge issues observed at higher current densities stem from the use of lithium metal and confirm the great performance of OTQC at higher rates.

**Figure 6:**
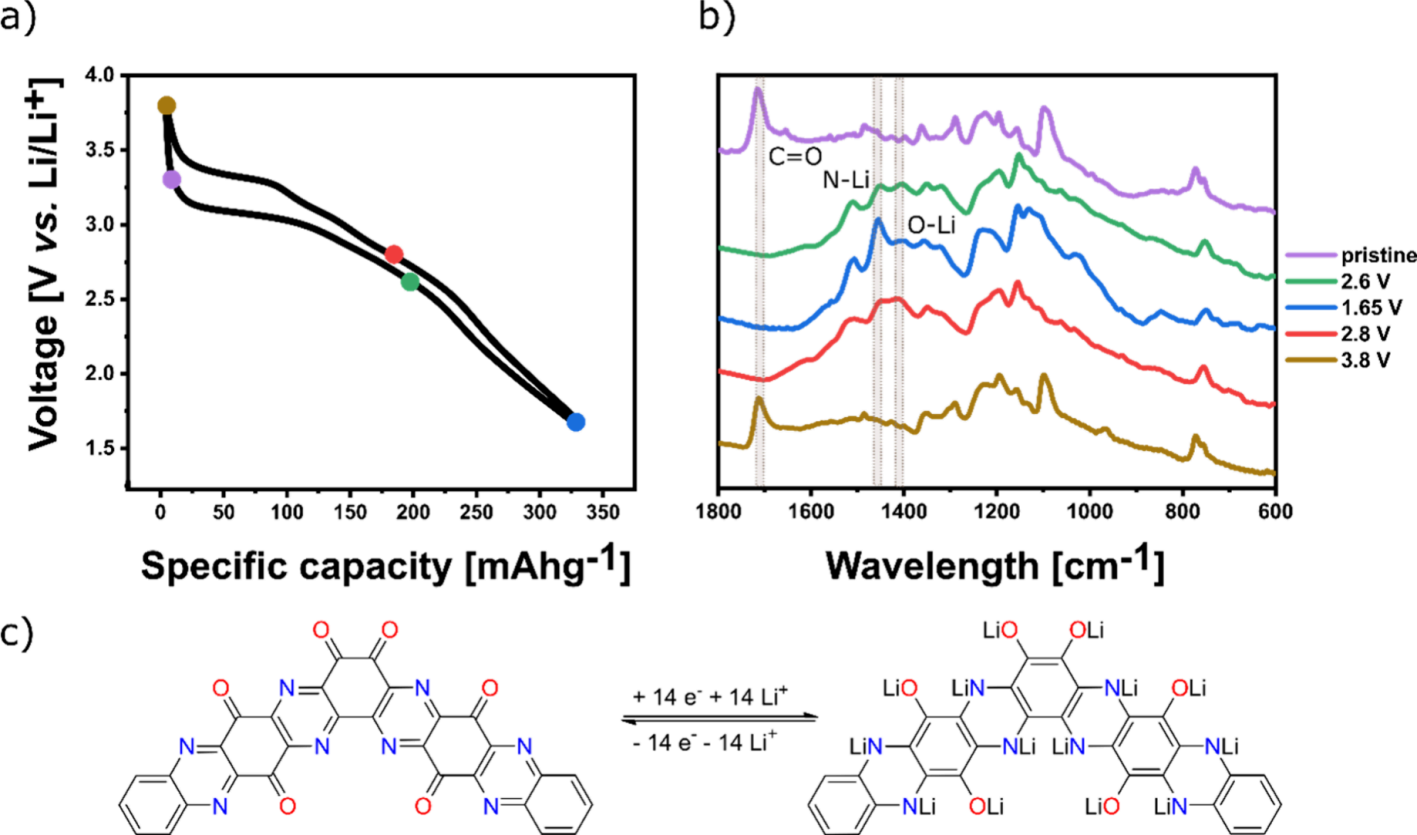
(a) Voltage profile showing the ex-situ FT-IR spectra sampling points. (b) Ex-situ FT-IR spectra of electrodes in different states of charge: pristine electrode submerged in the electrolyte (violet), discharged to 2.6 V (green), discharged to 1.65 V (blue), charged to 2.8 V (red), and charged to 3.8 V (brown). (c) Proposed redox mechanism of dimer OTQC in a lithium battery assuming complete reduction of pyrazine and quinone groups. The redox mechanism of the trimer OTQC is analogous to the dimer.

The electrochemical redox mechanism of the OTQC was studied with the use of ex-situ FT-IR measurements of the electrodes in different states of charge (Figure 6a,b). The electrodes (60 wt % OTQC, 30 wt % Printex XE2 carbon black, and 10 wt % PTFE) were taken out from the Swagelok cells after initial cycles, washed with DME, and measured in an argon atmosphere using KBr pellets in the transmittance mode. The results of the ex-situ FT-IR analysis were interpreted with the help of a literature review of similar reported structures.^21,22,39^ The FT-IR spectrum of the pristine electrode exhibits a strong peak at 1714 cm^–1^ attributed to the stretching vibrations of the carbonyl (C=O) group in the OTQC. Peaks attributed to the imine streching (C=N) vibrations in compounds containing pyrazine rings are found in broad region between 1600 and 1400 cm^–1^.^40,41^ The same region also encloses C=C stretching vibrations. In pristine OTQC several peaks are observed in that area, making the assignation of imine (C=N) vibrations unreliable. The carbonyl (C=O) peak disappears during discharging from 3.8 to 2.6 V (Figure 6a,b, green), which together with the emergence of the peaks at around 1404 and 1455 cm^–1^, attributed to O···Li and N···Li vibrations, respectively, signifies the simultaneous reduction of the carbonyl (C=O) and imine (C=N) groups in the OTQC (Figure 6c). Discharging from 2.6 to 1.65 V (Figure 6, blue) did not significantly change the intensity of the O···Li peak; in contrast, the peak associated with N···Li vibrations became better visible, indicating that the reduction of the imine groups contributed to the majority of the capacity. The FT-IR spectrum of the charged electrode at 2.8 V (Figure 6a,b, red) is very similar to the spectrum of the discharged electrode at 2.6 V (Figure 6a,b, green). Upon charging from 2.8 to 3.8 V (Figure 6a,b, brown), peaks visible in the pristine electrode reemerged, showing the high reversibility of the redox process.

**Figure 7:**
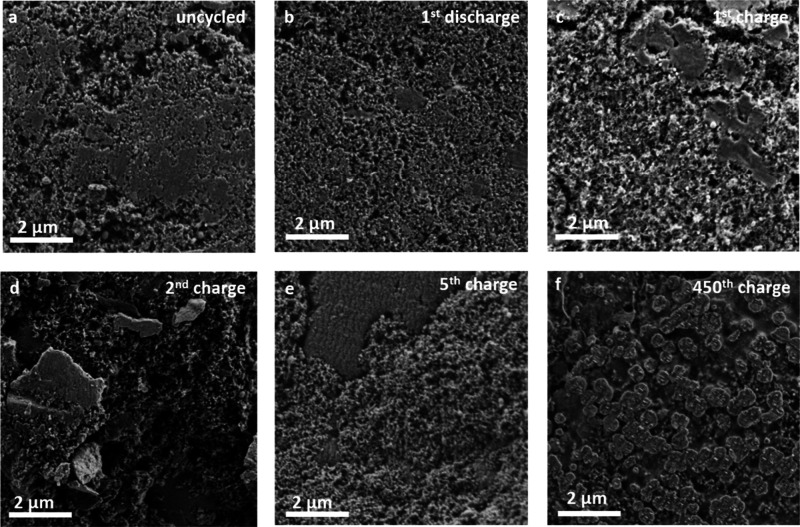
SEM images of charged/discharged OTQC electrodes after cycling in a Li–organic battery (cycled electrodes were washed with DME to remove the electrolyte residue). (a) Uncycled electrode. (b) Electrode after 1st discharge. (c) Electrode after 1st charge. (d) Electrode after 2nd charge. (e) Electrode after 5th charge. (f) Electrode after 450th charge.

To test the solubility of the OTQC, ex-situ UV–vis measurements of the electrodes in different states of charge were performed (Figure S7). Although the charged state (3.8 V) exhibits significantly higher absorption than discharged states (2.6 and 1.65 V), the absorption value does not necessarily correlate with the differences in solubility due to the possible changes in molar absorption coefficients between OTQC in different states of charge. Nevertheless, the results of UV–vis measurements show that the dissolution of the active material could present the main contribution to the capacity fading. OTQC composite electrodes were subjected to SEM imaging after 1, 2, 5, and 450 cycles. The pristine electrode shows relatively homogeneous surface morphology, with active particles evenly distributed through a porous carbon black matrix (Figure 7a). Overall, good contact between polymeric particles and carbon black is observed on the surface regardless of the charge cycle. However, there is cracking present around some particles in all of the cycled samples, which may be a consequence of volume expansions caused by swelling that occurs during cycling.^4^ No significant surface modifications or deposition products were observed in the initial cycle (Figure 7b,c). The integrality of the electrode is maintained even after 450 cycles, and interestingly, the surface of the electrode is observed to be covered by about 200–500 nm patches of deposits in addition to surface film-like formation observed at all the electrode surfaces (Figure 7f). The accumulation of deposits could be explained with the dissolution–redeposition mechanism already observed in other organic cathode materials,^32,42^ which can be influenced by the varying degree of solubility between OTQC discharged states in the electrolyte. Further research work is needed to identify the nature of the deposits and to thoroughly study the degradation mechanism(s), but this is beyond the scope of the present paper. SEM images of pristine OTQC powder are shown in Figure S8.

**Figure 8:**
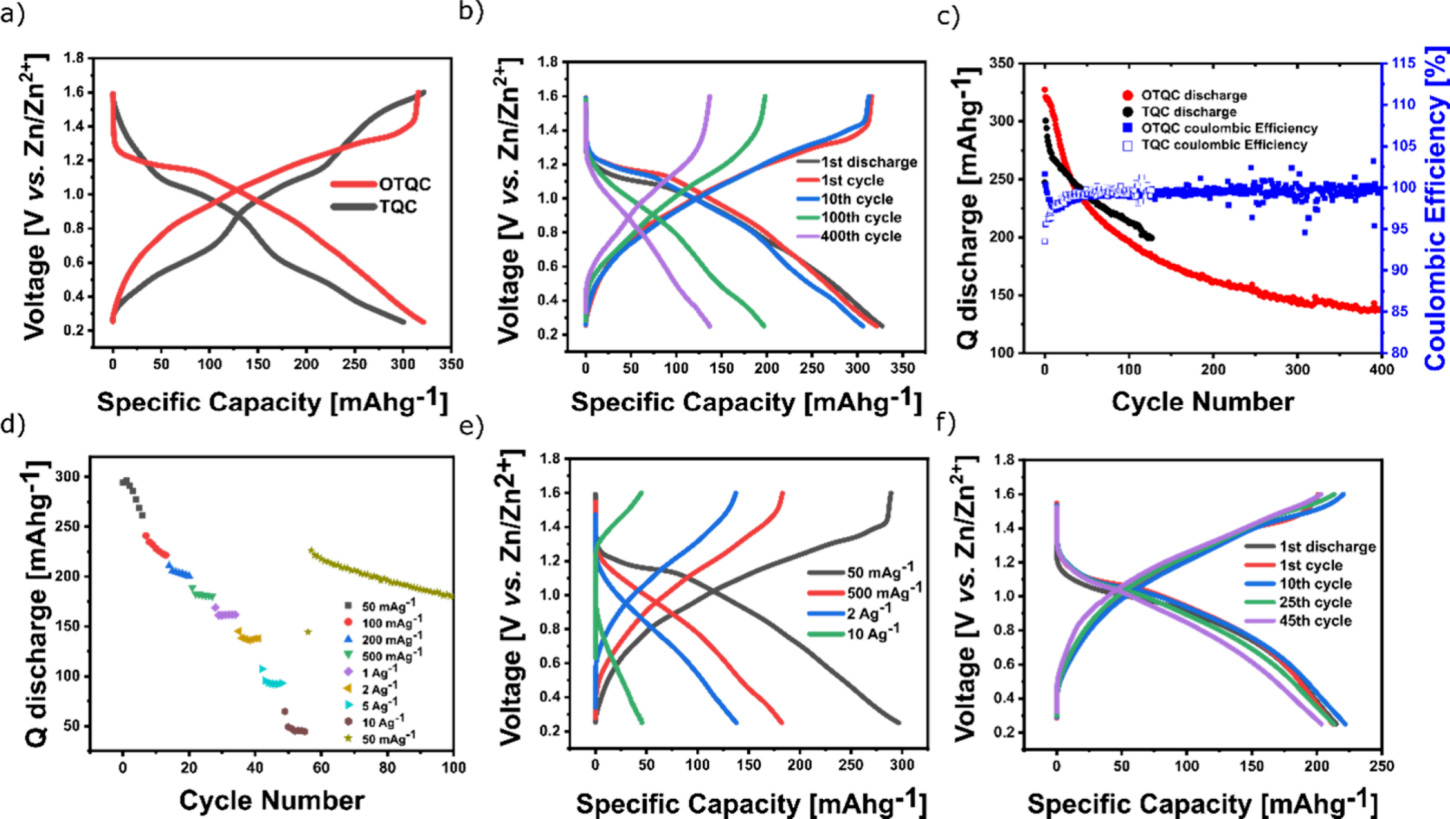
(a) Comparison of the galvanostatic charge/discharge curves between TQC (black, 2nd cycle) and OTQC (red, 2nd cycle) at 100 mA g^–1^ in 3 M ZnSO_4_. (b) Galvanostatic charge/discharge curves of OTQC at 100 mA g^–1^. (c) Cycling stability of OTQC (red) at 100 mA g^–1^ and TQC (black) at 50 mA g^–1^. (d, e) Rate performance of OTQC. (f) Galvanostatic charge/discharge curves of OTQC in 2.2 M Zn(OTf)_2_ in 70% PEG at 100 mA g^–1^.

The electrochemical performance of the synthesized OTQC was further assessed in a Zn–organic cell utilizing an aqueous 3 M ZnSO_4_ electrolyte. OTQC exhibited charge/discharge curves similar to those in the lithium system, with an initial plateau at around 1.2 V, which gradually transformed into a sloping curve (Figure 8a). In comparison with TQC, OTQC exhibited a slightly higher maximum capacity (326 mAh g^–1^ vs 301 mAh g^–1^) and a higher average discharge voltage (0.86 V vs 0.76 V). In contrast to the good cycling stability in the lithium system, OTQC showed significant capacity fading in the Zn battery, delivering a capacity retention of 42% after 400 cycles at 100 mA g^–1^ (Figure 8c). The rate performance test in the Zn system showed better reversibility without any overcharging at higher rates, as seen in the lithium system (Figure 8e). At the highest rate of 10 A g^–1^, the system reached a capacity of 47 mAh g^–1^ (21% of the capacity obtained at 100 mA g^–1^) (Figure 8d). In our recent report, we have shown that the use of less polar 2.2 M Zn(OTf)_2_ in 70% PEG electrolyte improved the cycling stability of a small organic cathode material TQD in a Zn–organic battery.^9,43^ The use of the aforementioned electrolyte decreased the specific capacity of OTQC, which reached a maximum value of 224 mAh g^–1^ but, on the other hand, drastically increased the cycling stability, reaching 91% capacity retention after 45 cycles at 100 mA g^–1^ (Figures 8f and S9).

**Figure 9:**
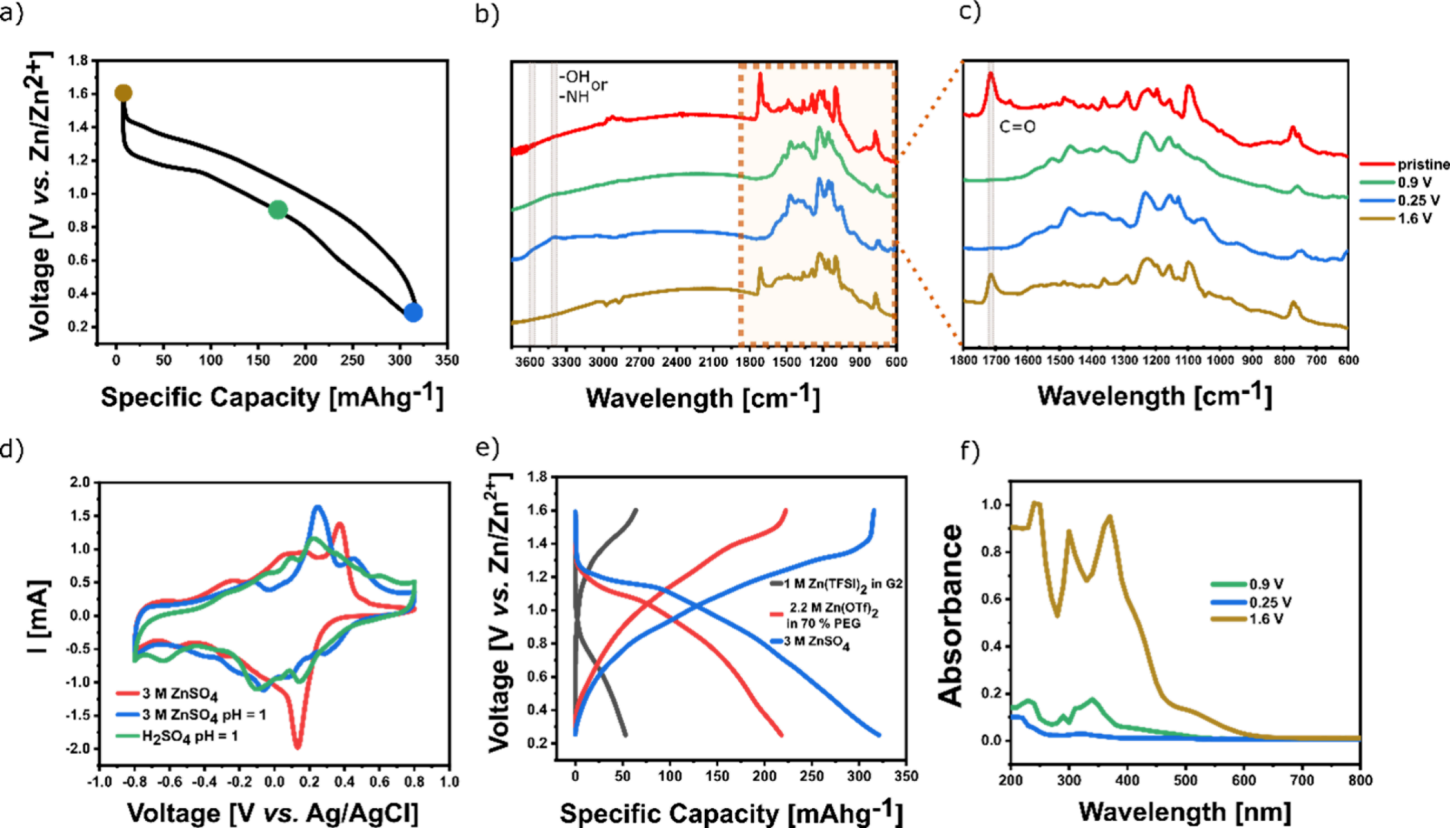
(a) Voltage profile showing the ex-situ FT-IR spectra sampling points. (b) Ex-situ FT-IR spectra of electrodes in different states of charge: pristine electrode (red), discharged to 0.9 V (green), discharged to 0.25 V (blue), and recharged to 1.6 V (brown). (c) Zoomed-in ex-situ FT-IR insert of electrodes in different states of charge. (d) CV curves of OTQC measured by a three-electrode system at 10 mV s^–1^ in 3 M ZnSO_4_ (pH = 3.7) (red), 3 M ZnSO_4_ + H_2_SO_4_ (pH = 1) (blue), and H_2_SO_4_ (pH = 1) green. (e) Galvanostatic charge/discharge curves of OTQC in different electrolytes including 1 M Zn(TFSI)_2_ in G2 (black, 5th cycle), 2.2 M Zn(OTf)_2_ in 70% PEG (red, 2nd cycle), and 3 M ZnSO_4_ (blue, 2nd cycle). (f) Ex-situ UV–vis spectrum of electrodes submerged in 3 M ZnSO_4_ at different states of charge: discharged to 0.9 V (green), discharged to 0.25 V (blue), and charged to 1.6 V (brown).

We employed several spectroscopic and electrochemical methods to determine the redox mechanism of OTQC in the OTQC-Zn battery (Figure 9). Ex-situ FT-IR measurements of the electrodes in different states of charge revealed that upon discharging to 0.9 V the peak at 1714 cm^–1^ associated with the carbonyl (C=O) stretching vibrations almost completely disappeared, which together with the absence of peaks above 3300 cm^–1^ associated with −NH and −OH vibrations suggests coordination with Zn^2+^ ions (Figure 9a–c, green). Discharge from 0.9 to 0.25 V showed the emergence of peaks at 3400–3600 cm^–1^ attributed to the −NH or −OH stretching vibrations, indicating H^+^ insertion (Figure 9a–c, blue). The peaks associated with H^+^ insertion were observed only in the OTQC-Zn battery and were absent in the water-free OTQC-Li battery (Figure S10). Recharging to 1.6 V exhibited reemergence of the peaks observed in the pristine electrode, signifying the reversibility of the redox process. The co-insertion redox mechanism of Zn^2+^ and H^+^ has already been reported for several other organic cathode materials.^9,26,27^ In contrast, recent reports suggest that the redox reaction of some organic cathode materials in aqueous zinc electrolytes involves only H^+^ insertion.^44,45^

To additionally confirm the possible Zn^2+^ and H^+^ co-insertion, we conducted three electrode cyclic voltammetry (CV) measurements employing the Pt counter electrode and the Ag/AgCl reference electrode. The measurements were performed using various aqueous electrolytes including 3 M ZnSO_4_ with a measured pH = 3.7, 3 M ZnSO_4_ + H_2_SO_4_ (pH = 1), H_2_SO_4_ (pH = 1), and 0.1 M ZnSO_4_ + H_2_SO_4_ (pH = 1) (Figures 9d and S11). Cyclic voltammetry in 3 M ZnSO_4_ revealed several peaks (Figure 9d, red), which roughly match the peaks obtained from galvanostatic cycling in the Zn-OTQC battery (Figure S11a). H_2_SO_4_ (pH = 1) electrolyte was used to simulate the H^+^ activity without the interference of Zn^2+^ ions, and the cyclic voltammogram showed electroactivity comparable with 3 M ZnSO_4_, which proves the possibility of H^+^ insertion into the OTQC (Figure 9d, green). The voltammogram of 3 M ZnSO_4_ + H_2_SO_4_ (pH = 1) exhibited peaks at different positions in comparison to either 3 M ZnSO_4_ or H_2_SO_4_ (pH = 1), indicating the possibility of Zn^2+^ and H^+^ co-insertion (Figure 9d, blue). The CV of the 0.1 M ZnSO_4_ + H_2_SO_4_ (pH = 1) electrolyte with an approximately equal concentration of Zn^2+^ and H^+^ ions showed almost identical features to the CV of H_2_SO_4_ (pH = 1), suggesting that the insertion of H^+^ ions into the OTQC is preferential if it is present in sufficient concentration (Figure S11b). Galvanostatic cycling in 1 M Zn(TFSI)_2_ in the G2 electrolyte was used to determine the feasibility of Zn^2+^ insertion without any interference from H^+^ ions.^26^ Despite substantially decreased capacity in comparison with 3 M ZnSO_4_ in H_2_O, galvanostatic cycling in the aprotic electrolyte proved the possibility of reversible Zn^2+^ insertion into the OTQC (Figure 9e, black). OTQC experienced significant capacity fading during galvanostatic cycling in the Zn battery using a 3 M ZnSO_4_ electrolyte. Ex-situ UV–vis spectra of the electrodes in different states of charge submerged in 3 M ZnSO_4_ were used to study the dissolution behavior of OTQC (Figure 9f). The results showed that OTQC is partially soluble in all states of charge. The choice of appropriate voltage window was shown to have a great effect on the cycling stability of small organic cathode materials.^9,26^ The use of a narrower window between 0.25 and 0.9 V decreased the specific capacity but, on the other hand, greatly improved the cycling stability (Figure S12).

**Figure 10:**
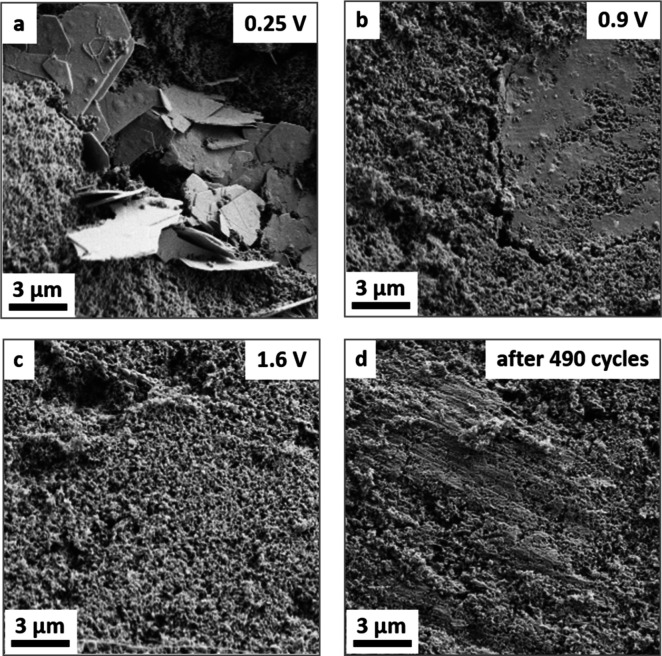
SEM images of OTQC electrodes stopped at different voltages in Zn: (a) 0.25 V after 5 cycles, (b) 0.9 V after 5 cycles, (c) 1.6 V after 5 cycles, and (d) 1.6 V after 490 charge/discharge cycles.

Structure evolution of OTQC at different states of charge was performed on composite electrodes cycled in 3 M ZnSO_4_ (Figure 10). At the lowest voltage, flakes several micrometers in size were observed throughout the surface of discharged electrodes (Figure 10a). These deposits are most likely salt byproducts formed due to the increased local pH after H^+^ insertion into the OTQC electrode, as was reported recently.^26,27^ To support this claim, EDS mapping was performed (Figure S13). The presence of sulfur and zinc was detectable on the surface, with areas of increased Zn, S, and O concentrations matching, indicating the formation of inorganic deposits. The findings further support the H^+^ insertion as seen from the ex-situ FT-IR spectrum of the discharged electrode. Similarly to electrodes cycled in Li-based electrolytes, the overall structural integrity of the electrode seems to be maintained even after prolonged cycling, with no detectable deterioration of the contact between active particles and carbon black (Figure 10d).

## 3 Conclusions

In summary, we presented a novel high-performance organic cathode material obtained with a facile two-step synthesis from commercially available precursors. In the OTQC-Li battery, newly synthesized OTQC material delivered a specific capacity of 327 mAh g^–1^ at 50 mA g^–1^ with an average voltage of 2.63 V, corresponding to one of the highest reported energy densities for redox-active organics of 860 Wh kg^–1^ on the material level Furthermore, the material demonstrated excellent cycling stability having capacity retention of 82% after 400 cycles at 50 mA g^–1^, which decreased to 77% after 800 cycles at a high current of 1 A g^–1^. By excluding the limitations of a Li metal anode, a symmetric cell approach and OTQC-LTO cell testing enabled finding excellent cycling stability of OTQC electrodes at high current densities. Increased performance in comparison with TQC was also observed in the OTQC-Zn battery where it exhibited a specific capacity of 326 mAh g^–1^ with an average voltage of 0.86 V at 100 mA g^–1^ but showed worse cycling stability reaching 42% after 400 cycles. We believe that the high theoretical capacity of the OTQC material and the excellent practically demonstrated electrochemical stability of the corresponding electrodes present definite motivation for further improvements of synthesis approaches and electrode engineering efforts on the path to reaching reliable high-energy battery systems.

## 4 Experimental Section

### Synthesis of Tetraquinoxalinecathechol (TQC)

TQC was synthesized as previously reported.^35^ A mixture containing 2,3-diaminophenazine (1.58 g, 7.52 mmol) and sodium rhodizonate (0.70 g, 3.27 mmol) in 50 mL of deoxygenated glacial acetic acid was heated under an inert atmosphere for 48 h at 120 °C (Figure 2a). Afterward, the mixture was allowed to cool to the room temperature and filtered. The solid product was washed with glacial acetic acid, water, ethanol, and acetone. To further purify the product, it was subjected to 24 h Soxhelt extraction with ethanol. Upon drying at 80 °C overnight, 1.66 g of TQC was obtained as a black powder (98% yield based on the formation of pure catechol TQC).

### Synthesis of Oxidized Tetraquinoxalinecathechol (OTQC)

TQC powder (0.7 g) was suspended in 150 mL of acetic acid by using an ultrasonic bath. After the addition of K_2_Cr_2_O_7_ (5.95 g, 20.25 mmol) to the suspension, the mixture was heated at 100 °C for 6 h under an inert atmosphere. Afterward, the mixture was allowed to cool to room temperature, filtered, and washed with water, acetone, and ethanol. The material was further purified using two sequential 16 h Soxhlet extractions with water and ethanol. The obtained product was dried at 80 °C overnight, yielding 0.38 g of OTQC (49% yield based on the formation of pure dimer OTQC).

## Supporting Information Available

Material characterization, electrode preparation and battery assembly, electrochemical measurements; ex-situ FT-IR and UV–vis characterization of electrodes, cycling of OTQC in a wider voltage window, capacity contribution of Printex XE2 carbon additive, electrolyte stability window, cycling in LP30 electrolyte, charge/discharge curves of Li-LTO battery, ex-situ UV–vis spectra of OTQC electrodes, SEM images, comparison of cycling stability of QTQC in two zinc electrolytes, comparison of FT-IR spectra of discharged OTQC electrodes from Li and Zn batteries, CV curves at different pHs, comparison of cycling stability of the Zn-OTQC battery in two voltage windows, EDS elemental mapping of the cycled electrode, table of measured elemental analysis vs theoretical calculations, table comparing the performance of reported small organic cathode materials in Li– and Zn–organic batteries (PDF)
