# Supplementary material for: Design of Organic Cathode Material Based on Quinone and Pyrazine Motifs for Rechargeable Lithium and Zinc Batteries
Source: ACS Appl Mater Interfaces. 2024 Mar 21;16(13):16029–39. doi: 10.1021/acsami.3c16038 (PMC10995900; doi:10.1021/acsami.3c16038)
Supplement: Supplementary file 1 — am3c16038_si_001.pdf [file am3c16038_si_001.pdf]

SUPPORTING INFORMATION

Design of organic cathode material based on quinone and pyrazine motifs for rechargeable lithium and zinc batteries

Svit Menart[1,2], Olivera Lužanin[1,2], Klemen Pirnat[1*], David Pahovnik[1], Jože Moškon[1] and Robert Dominko[1,2,3]

[1]*National Institute of Chemistry, Hajdrihova 19, 1001 Ljubljana, Slovenia*

[2]*Faculty of Chemistry and Chemical Technology, University of Ljubljana, Večna pot 113, 1000 Ljubljana, Slovenia*

[3]*ALISTORE-European Research Institute, 33 rue Saint-Leu, 80039 Amiens, France*

*Email: klemen.pirnat@ki.si

Material characterization

^{13}C MAS NMR spectra were recorded on Avance Neo 400 MHz spectrometer (Bruker) equipped with a 4 mm CP-MAS probe with ^{1}H-^{19}F and ^{31}P-^{15}N coils. The sample rotation frequency was 10 kHz and the relaxation delay was set to 5s. Chemical shifts are given in ppm relative to the tetramethyl silane ($SiMe_4$) standard. FT-IR spectroscopy was recorded on IFS66/S (Bruker) using KBr pellets in the wavelength range of 600-4000 cm^{-1}. Mass spectrometry measurements were recorded on UltrafleXtreme MALDI-TOF (Bruker Daltonik) mass spectrometers. The samples were first mixed with the matrix dithranol in an agate mortar. The solid mixture was then transferred to the MALDI plate using a spatula and the reflective positive ion mode was used to acquire the mass spectra. Calibration was performed externally using a poly(methyl methacrylate) standard (MALDI validation set PMMA, Fluka Analytical).

Electrochemical measurements

All electrochemical measurements were carried out using potentiostat/galvanostat VMP3 (Bio-Logic, France) at room temperature (25 °C).

Lithium battery

The working electrode was prepared by mixing 60 wt. % of the active material, 30 wt. % of Printex XE2 carbon black, and 10 wt. % of polytetrafluoroethylene (PTFE) binder (60 wt. % water dispersion, Aldrich) in 2-propanol. The mixture was ball milled in a planetary ball mill (Retsch PM100) at 300 rpm for 30 min in an ambient atmosphere. The obtained slurry was rolled into a thin film, pressed onto an Al-mesh (mesh size 100) current collector, and cut into circular discs (ϕ = 12 mm), which were afterward dried at 50 °C for 1 day. For ex-situ FT-IR measurements, the slurry was rolled on glass and cut into free-standing electrodes (ϕ = 12 mm).

Stainless steel Swagelok-type battery cells were assembled in an argon-filled glovebox (O_2 < 1 ppm, H_2O < 1 ppm) by separating working electrodes and lithium foil discs (ϕ = 12 mm) with 2 pieces (ϕ = 13 mm) of Celgard 2320 separators wetted with 3 drops of 1 M LiTFSI in 1:1 (v/v) DOL and DME.

Zinc battery

Working electrodes were prepared by mixing 60 wt. % of the active material, 30 wt. % of Printex XE2 carbon black, and 10 wt. % of polytetrafluoroethylene (PTFE) binder (60 wt. % water dispersion, Aldrich) in 2-propanol. The mixture was ball milled in a planetary ball mill (Retsch PM100) at 300 rpm for 30 min in an ambient atmosphere. The obtained slurry was rolled into a thin film on glass and cut into circular discs (ϕ = 10 mm), which were afterward dried at 50 °C for 1 day to obtain free-standing electrodes. Stainless steel Swagelok-type battery cells were assembled in ambient atmosphere* by separating free-standing cathode electrodes and zinc foil discs (ϕ = 10 mm) with 2 pieces (ϕ = 13 mm) of glass fiber separator (Whatman GF/A) wetted with 3 drops of 3 M $ZnSO_4$, 2.2 M $Zn(OTf)_2$ in 70 % PEG or 1 M $Zn(TFSI)_2$ in G2 electrolyte. 3 M $ZnSO_4$ electrolyte was prepared by dissolving an appropriate amount of zinc sulfate heptahydrate ($ZnSO_4 \cdot 7H_2O$) in water. 2.2 M $Zn(OTf)_2$ in 70 wt. % PEG was prepared according to the literature procedure[1] by dissolving 2 mmol of zinc triflate ($Zn(OTf)_2$) in 0.7 g of polyethylene glycol 400 (PEG 400) and 0.3 g of water. 1 M $Zn(TFSI)_2$ in G2 was prepared by dissolving zinc bis(trifluoromethylsulfonyl)imide ($Zn(TFSI)_2$) in (G2) solvent.

*Battery cells with 1 M $Zn(TFSI)_2$ in G2 electrolyte were assembled in an argon filled glovebox (O_2 < 1 ppm, H_2O < 1 ppm).

OTQC symmetric cell

One OTQC electrode was discharged with 20 mAg^{-1} down to 1.65 V and the voltage was held for 12 h. Disassembly was performed in an inert atmosphere (no washing in between). The discharged electrode was paired with a pristine OTQC electrode. One Celgard 2320 was used as a separator, with an additional three drops of 1 M LiTFSI in 1:1 (v/v) DOL and DME electrolyte. The cell was cycled at 20 mAg^{-1} for 5 cycles first and then subjected to high current charging/discharging.

OTQC-LTO battery

Lithium-titanate (LTO) electrode was prepared by mixing 80 wt. % of the active material, 10 wt. % of C65 carbon black, and 10 wt. % of polyvinylidene fluoride (PVDF) binder in *N*-methyl-2-pyrrolidone (NMP). The mixture was ball milled in a planetary ball mill (Retsch PM100) at 300 rpm for 30 min in an ambient atmosphere. Obtained slurry was doctor blade coated onto a copper current collector and cut into circular discs (ϕ = 12 mm), which were afterward dried at 100 °C under vacuum for 1 day. Obtained electrodes were pressed with 1 ton/cm^2 of pressure and dried for an additional 3 h at 100 °C under vacuum.

Lithiated LTO anodes were harvested in the glovebox from the stainless steel Swagelok LTO-Li battery, where the LTO electrode was discharged at 50 mAg^{-1} to approximately 80 % of the maximum obtained specific capacity. The lithiated LTO electrode was afterward paired with the pristine OTQC electrode and separated with a Celgard 2320 separator, with an additional three drops of 1 M LiTFSI in a 1:1 (v/v) DOL and DME electrolyte. The obtained cell was cycled in different voltage windows corresponding to the cycling of OTQC-Li battery (1.65 V

– 3.8 V), taking into account the voltage hysteresis of LTO material at different current densities.

Three-electrode measurements

Three-electrode measurements were done using Ag/AgCl electrode and platinum coil as reference and counter electrode, respectively. The working electrode was obtained by drop-casting slurry composed of 60 wt. % of OTQC, 30 wt. % of Super C65 carbon black and 10 wt. % of polyvinylidene fluoride (PVDF) solution (10 mg/mL) in *N*-methyl-2-pyrrolidone (NMP) on a glassy carbon electrode and afterward drying at 50 °C for 2h. A continuous flow of nitrogen ensured an oxygen-free environment for the measurements. Different concentrations of $ZnSO_4$ electrolytes were prepared by dissolving an appropriate amount of zinc sulfate heptahydrate ($ZnSO_4 \cdot 7H_2O$) in water. 3 M $ZnSO_4$ + H_2SO_4 (pH = 1) electrolyte was prepared by the addition of H_2SO_4 into 3 M $ZnSO_4$ until the pH meter (827 pH Lab, Metrohm) measured pH = 1. 0.1 M $ZnSO_4$ + H_2SO_4 (pH = 1) electrolyte was prepared by the addition of H_2SO_4 into 0.1 M $ZnSO_4$ until the pH meter (827 pH Lab, Metrohm) measured pH = 1. H_2SO_4 (pH = 1) and H_2SO_4 (pH = 3.3) electrolyte solutions were obtained by the addition of H_2SO_4 into water until the appropriate pH meter (827 pH Lab, Metrohm) measured value.

Computational calculations

Density functional theory (DFT) computations were carried out using the B3LYP hybrid density functional with a 6-31G* basis set as implemented in the Spartan'14 program.

Ex-situ characterizations

Ex-situ FT-IR characterization of Li-OTQC electrodes: FT-IR spectroscopy was recorded on IFS66/S (Bruker) using KBr pellets in the wavelength range of 600-3800 cm^{-1}. OTQC cathodes were harvested from Swagelok-type cells using self-standing electrodes obtained by the process described in electrochemical measurements. The cathode electrodes were obtained by disassembling the batteries in different states of charge in an argon-filled glovebox and rinsed with DME solvent. Washed electrodes were afterward dried under vacuum and mixed with KBr to form the pellet.

Ex-situ FT-IR characterization of Zn-OTQC electrodes: FT-IR spectroscopy was recorded on IFS66/S (Bruker) using KBr pellets in the wavelength range of 600-3800 cm^{-1}. OTQC cathodes were harvested from Swagelok-type cells described in electrochemical measurements with an additional hydrophilic polytetrafluoroethylene (PTFE) membrane separator (0.2 μm pore size, Omnipore), which avoided the contamination with glassy fibers. The cathode electrodes were obtained by disassembling the batteries in different states of charge in an argon-filled glovebox and rinsing them with water to remove the residual electrolyte. Washed electrodes were afterward dried at 50 °C and mixed with KBr to form the pellet.

Ex-situ UV-VIS characterization of Zn-OTQC electrodes: Ex-situ UV-VIS measurements were carried out using LAMBDA 950 (PerkinElmer) with 3 M $ZnSO_4$ as a blank solution

reference in a wavelength range of 200-800 nm. OTQC cathodes after 5 cycles were stopped at different states of charge and harvested from disassembled batteries in an argon-filled glovebox and submerged in 3 M $ZnSO_4$ solution for 7 days.

Ex-situ UV-VIS characterization of Li-OTQC electrodes: Ex-situ UV-VIS measurements were carried out using LAMBDA 950 (PerkinElmer) with 1 M LiTFSI in 1:1 (v/v) DOL and DME as a blank solution reference in the wavelength range of 200-800 nm. OTQC cathodes after 5 cycles were stopped at different states of charge and harvested from disassembled batteries in an argon-filled glovebox and submerged in 1 M LiTFSI in 1:1 (v/v) DOL and DME solution for 1 month.

Scanning Electron Microscopy (SEM): Imaging was conducted on FE-SEM Supra 35 VP Carl Zeiss, at an accelerating voltage of 1.5 kV, with the use of an SE2 detector. Elemental mapping was performed with the use of an EDS detector at an accelerating voltage of 20 kV.

Prior to imaging, electrodes from the Li-OTQC battery were charged to 3.8 V or discharged to 1.65 V (50 mA g^{-1}). Cells were disassembled inside an Ar-filled glovebox and washed three times in 2 ml of fresh DME. Electrodes were then left to dry for two hours. Transfer to the SEM was performed in a vacuum, to avoid any potential material degradation.

Electrodes from the Zn-OTQC battery were taken out from Swagelok-type cells as explained in ex-situ FT-IR characterization. Transfer to the SEM was performed in a vacuum, to avoid any potential material degradation.

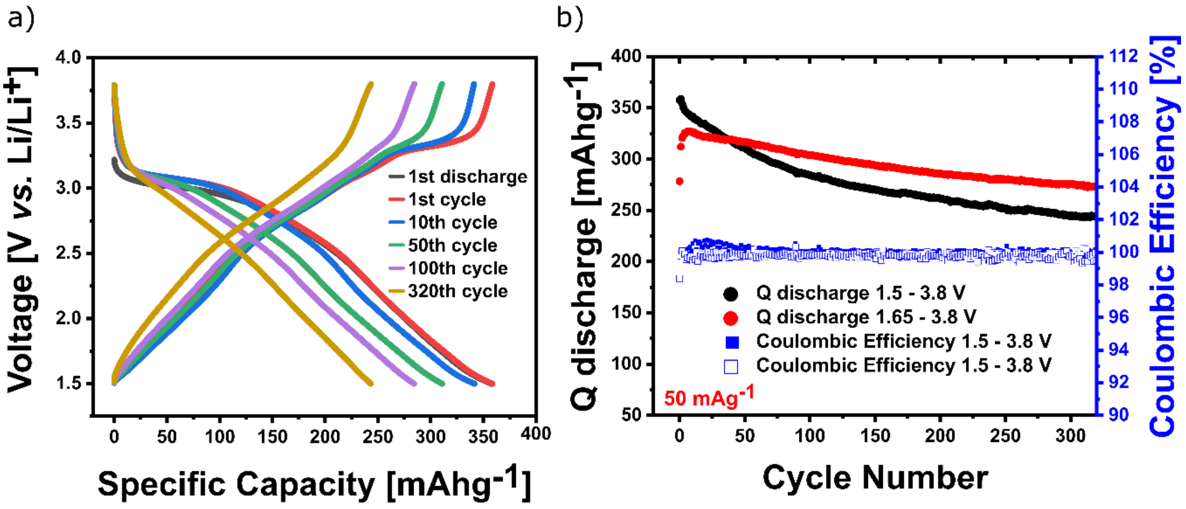

Figure S1: a) Charge/discharge curves of OTQC in Li-organic battery in the wider voltage range of 1.5 – 3.8 V at 50 mAg^{-1}. b) Comparison of cycling stability between OTQC in the wider voltage range of 1.5 – 3.8 V (black) and narrower voltage window of 1.65 – 3.8 V (red) at 50 mAg^{-1}. OTQC showed higher maximum capacity but worse cycling stability reaching 68 % capacity retention after 320 cycles.

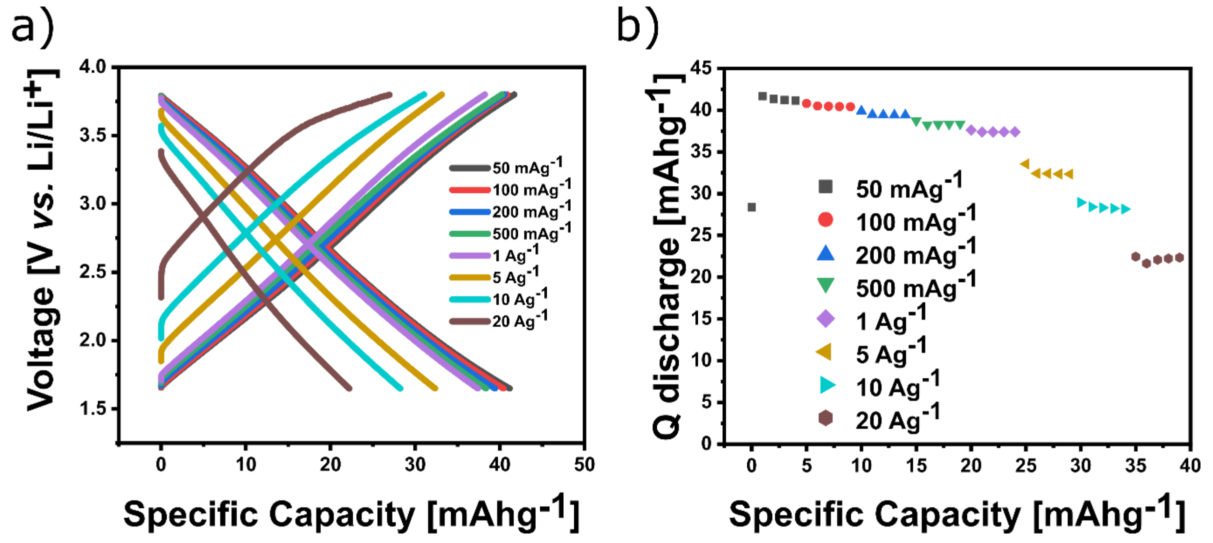

Figure S2: Capacity contribution measurement of Printex XE2 carbon additive using blank electrode (Printex XE2:PTFE = 70:30 wt%) in 1 M LiTFSI in 1:1 (v/v) DOL and DME electrolyte. a) Charge/discharge curves and b) Discharge capacity at different current rates from 50 mAg^{-1} to 20 Ag^{-1}.

The capacity contribution of Printex XE2 to the measured capacity of OTQC at 50 mAg^{-1} has been estimated using following equation:

$$C_{OTQC} = C_{meas} - \frac{W_{Printex\ XE2}}{W_{OTQC}} C_{Printex\ XE2} = 306\ mAhg^{-1}$$

C_{OTQC}…real specific capacity of OTQC ($mAhg^{-1}$)
C_{meas}…measured specific capacity (327 $mAhg^{-1}$)
$w_{Printex\ XE2}$…wt. % of Printex XE2 in the electrode (30%)
w_{OTQC}… wt. % of OTQC in electrode (60%)
$C_{Printex\ XE2}$… specific capacity of Printex XE2 (42 $mAhg^{-1}$)

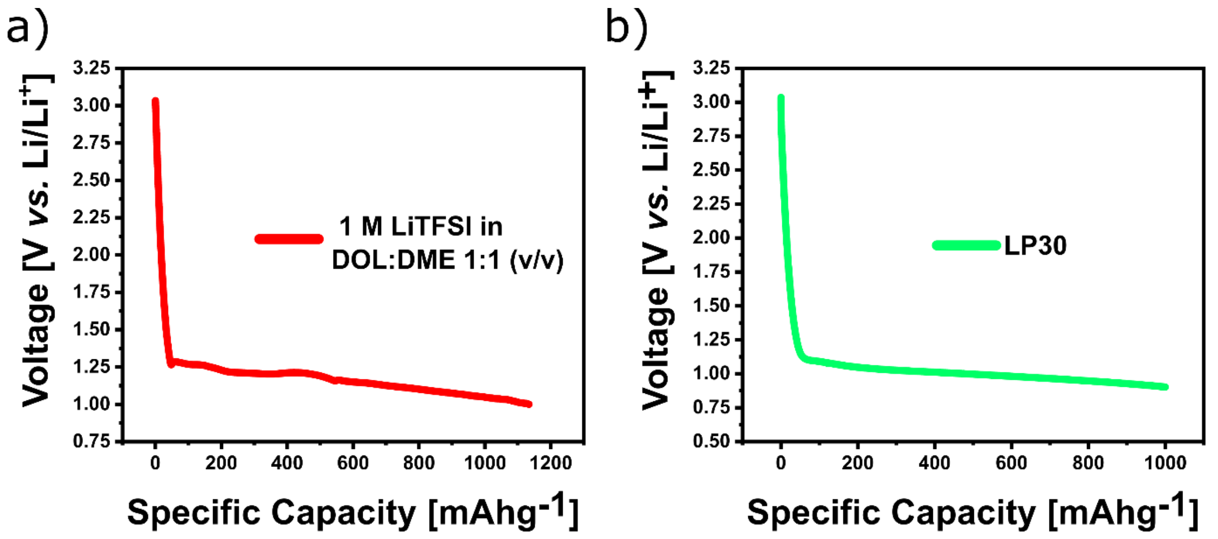

Figure S3: Electrolyte stability measurement using blank electrode (Printex XE2:PTFE = 70:30 wt%) in a) 1 M LiTFSI in 1:1 (v/v) DOL and DME electrolyte, showing electrolyte degradation below 1.3 V, and b) LP30 electrolyte, showing electrolyte degradation below 1.15

V.

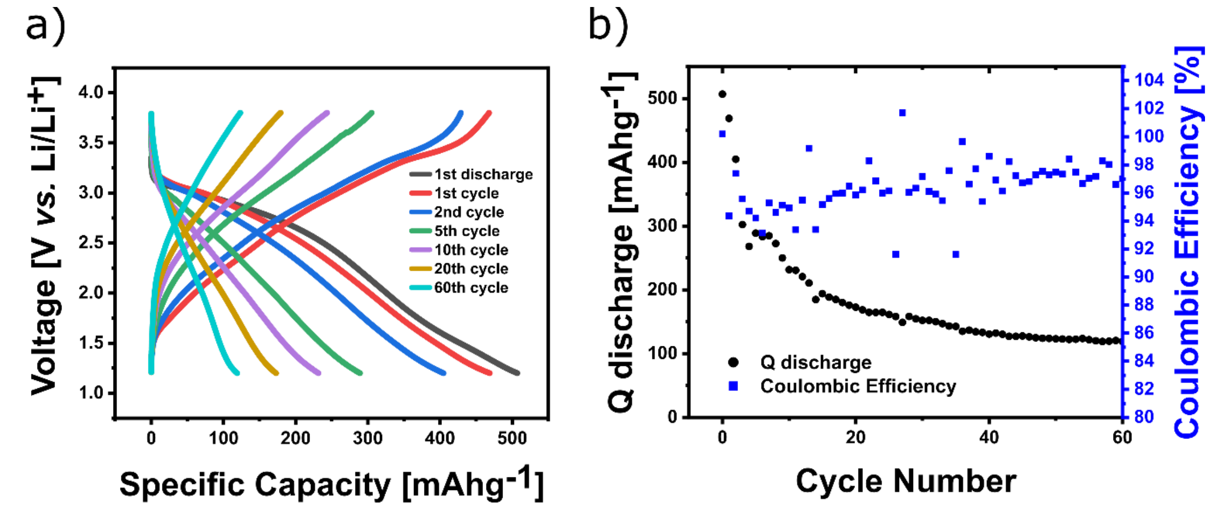

Figure S4: a) Charge/discharge curves of OTQC LP30 electrolyte utilizing the wider voltage range of 1.2 – 3.8 V at 50 mAg^{-1}. b) Cycling stability of OTQC in LP30 electrolyte.

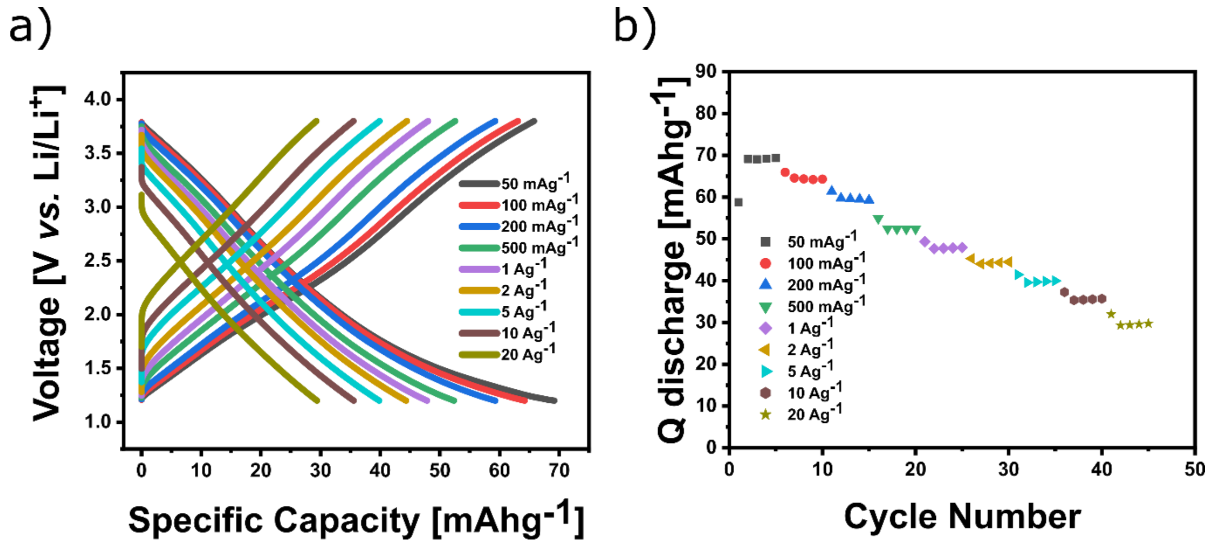

Figure S5: Capacity contribution measurement of Printex XE2 carbon additive using blank electrode (Printex XE2:PTFE = 70:30 wt%) in LP30 electrolyte. a) Charge/discharge curves and b) Discharge capacity at different current rates from 50 mAg^{-1} to 20 Ag^{-1}.

The capacity contribution of Printex XE2 to the measured capacity of OTQC at 50 mAg^{-1} has been estimated using following equation:

$$C_{OTQC} = C_{meas} - \frac{w_{Printex\ XE2}}{w_{OTQC}} C_{Printex\ XE2} = 473\ mAhg^{-1}$$

C_{OTQC}…real specific capacity of OTQC ($mAhg^{-1}$)
C_{meas}…measured specific capacity (507 $mAhg^{-1}$)
$w_{Printex\ XE2}$…wt. % of Printex XE2 in the electrode (30%)

w_{OTQC}… wt. % of OTQC in electrode (60%)
$C_{Printex\ XE2}$… specific capacity of Printex XE2 (68 mAhg^{-1})

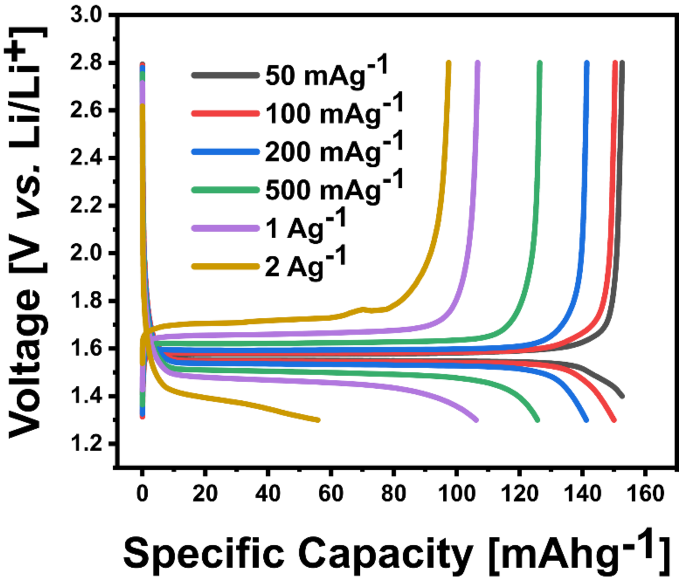

Figure S6: Charge/discharge curves of LTO in LTO-Li battery. At a high current density of 2 Ag^{-1} (brown) overcharging similar to the OTQC-Li battery is observed.

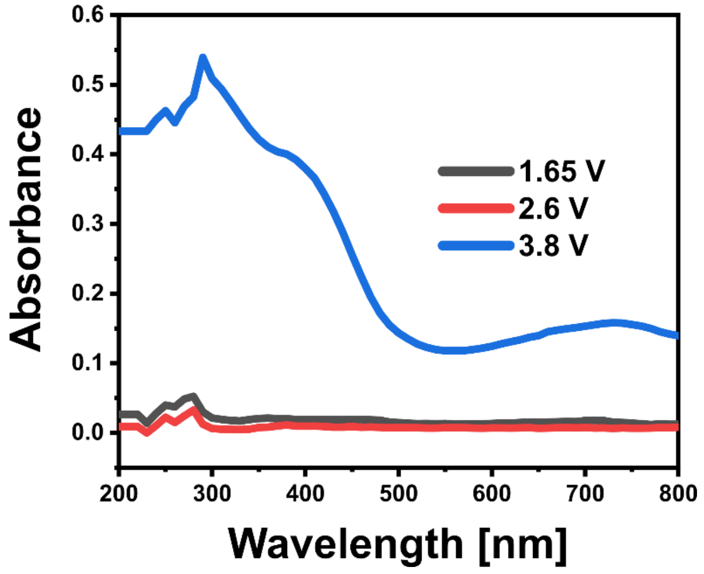

Figure S7: Ex-situ UV-VIS spectra of OTQC electrodes at different states of charge (voltage) submerged in 1 M LiTFSI in 1:1 (v/v) DOL and DME electrolyte: charged to 3.8 V (blue), discharged to 2.6 V (red), and discharged to 1.65 V (black). The electrodes were submerged

into 3 mL of electrolyte for 3 weeks before measuring.

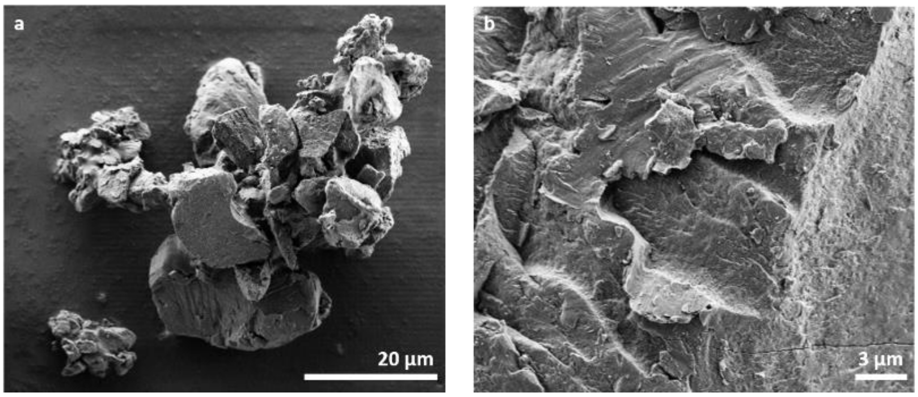

Figure S8: SEM images of the pristine OTQC powder at different magnifications. The SEM analysis of the OTQC powder revealed agglomerates of irregularly shaped particles spanning a range of sizes, exhibiting a compact structure. a) Agglomerate size 30×60 µm and b) Zoomed surface area 26×23 µm.

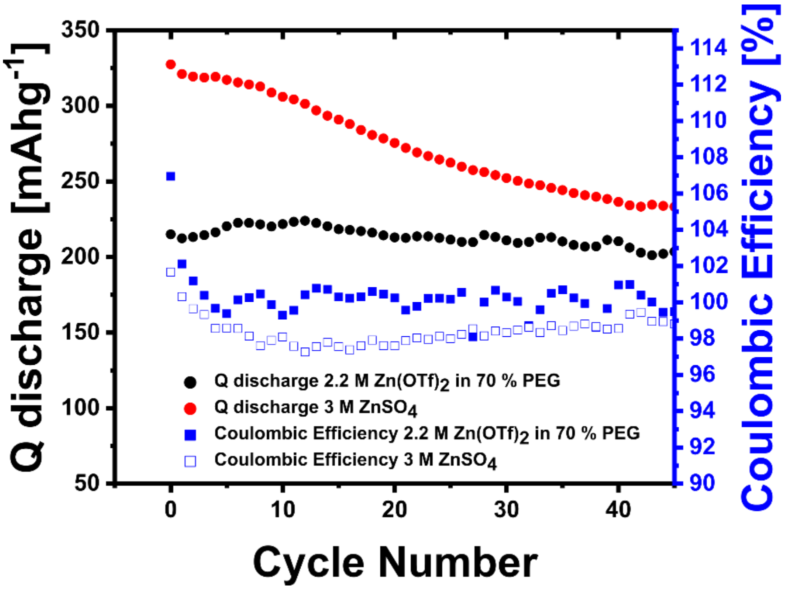

Figure S9: Comparison of cycling stability of OTQC in 2.2 M $Zn(OTf)_2$ in 70 % PEG[1] (black) and OTQC in 3 M $ZnSO_4$ (red) electrolyte at 100 mAg^{-1}.

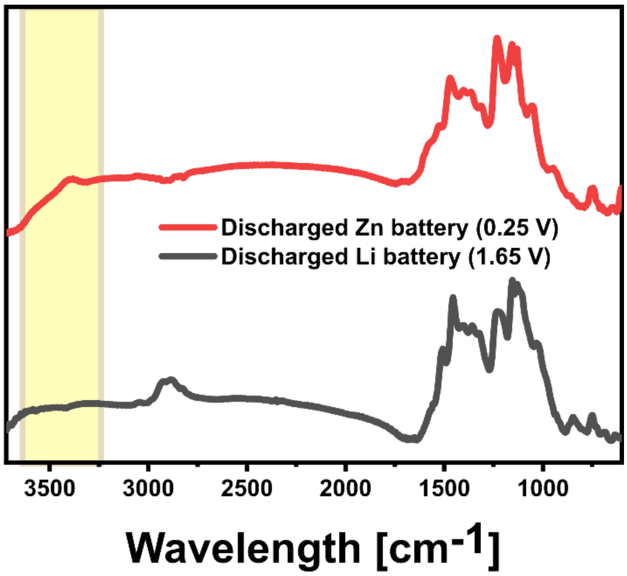

Figure S10: Comparison of FT-IR spectra between the discharged electrode in OTQC-Zn battery (red) and OTQC-Li battery (black). FT-IR spectrum of OTQC-Li electrode shows an absence of peaks above 3200 cm^{-1} associated with -OH or -NH vibrations.

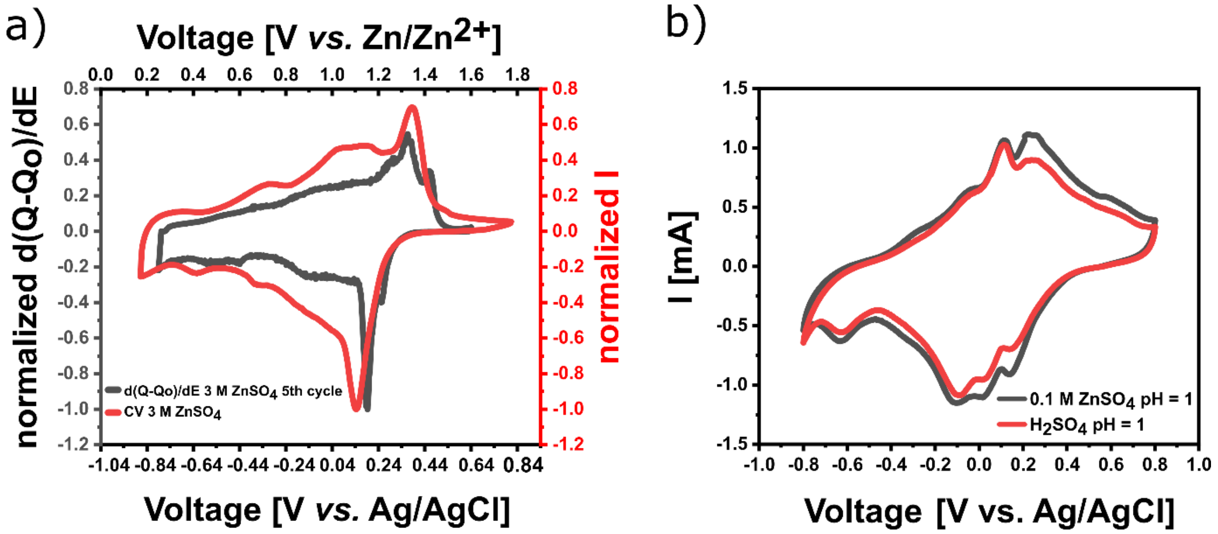

Figure S11: a) Comparison between d($Q - Q_0$)/dE curve obtained from the 5th galvanostatic cycle in Zn-battery using 3 M $ZnSO_4$ electrolyte (black) and a CV curve measured by a three-electrode system at 10 mV s^{-1} and shifted according to Zn/Zn^{2+} *vs.* Ag/AgCl = −0.957 V *vs.* Ag/AgCl. b) Comparison of CV between OTQC in 0.1 M $ZnSO_4$ + H_2SO_4 (pH = 1) (black) and H_2SO_4 (pH = 1) (red) electrolyte.

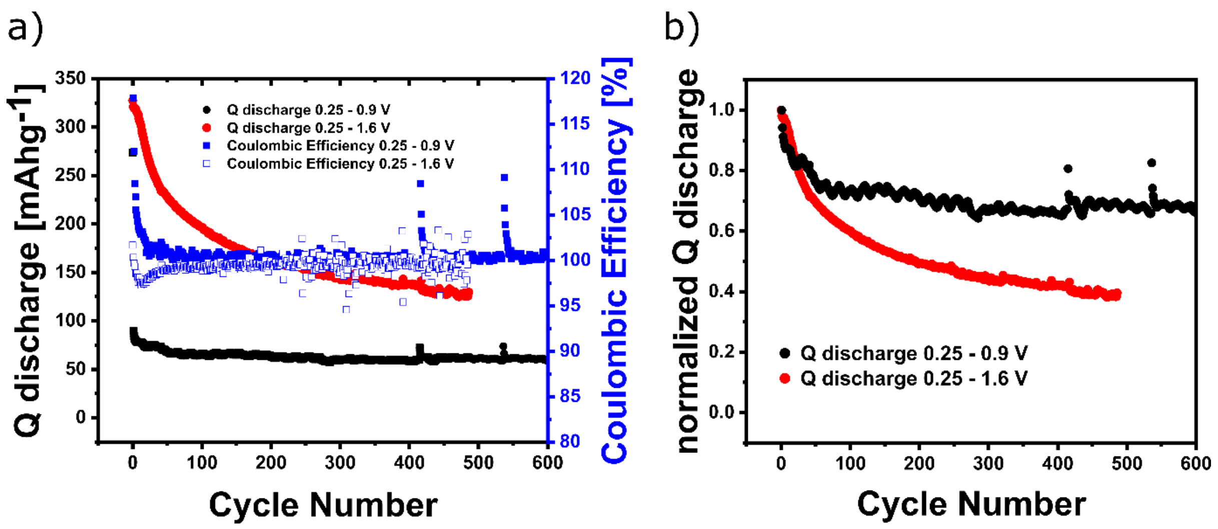

Figure S12: a) Comparison of cycling performance of OTQC-Zn battery in the voltage window between 0.25 – 0.9 V (black) and 0.25 – 1.6 V (red). In the narrower voltage window of 0.25 – 0.9 V OTQC exhibits lower capacity but higher capacity retention of 77 % after 600 cycles relative to the 10th cycle. b) Comparison of normalized discharge capacities between 0.25 – 0.9 V (black) and 0.25 – 1.6 V (red).

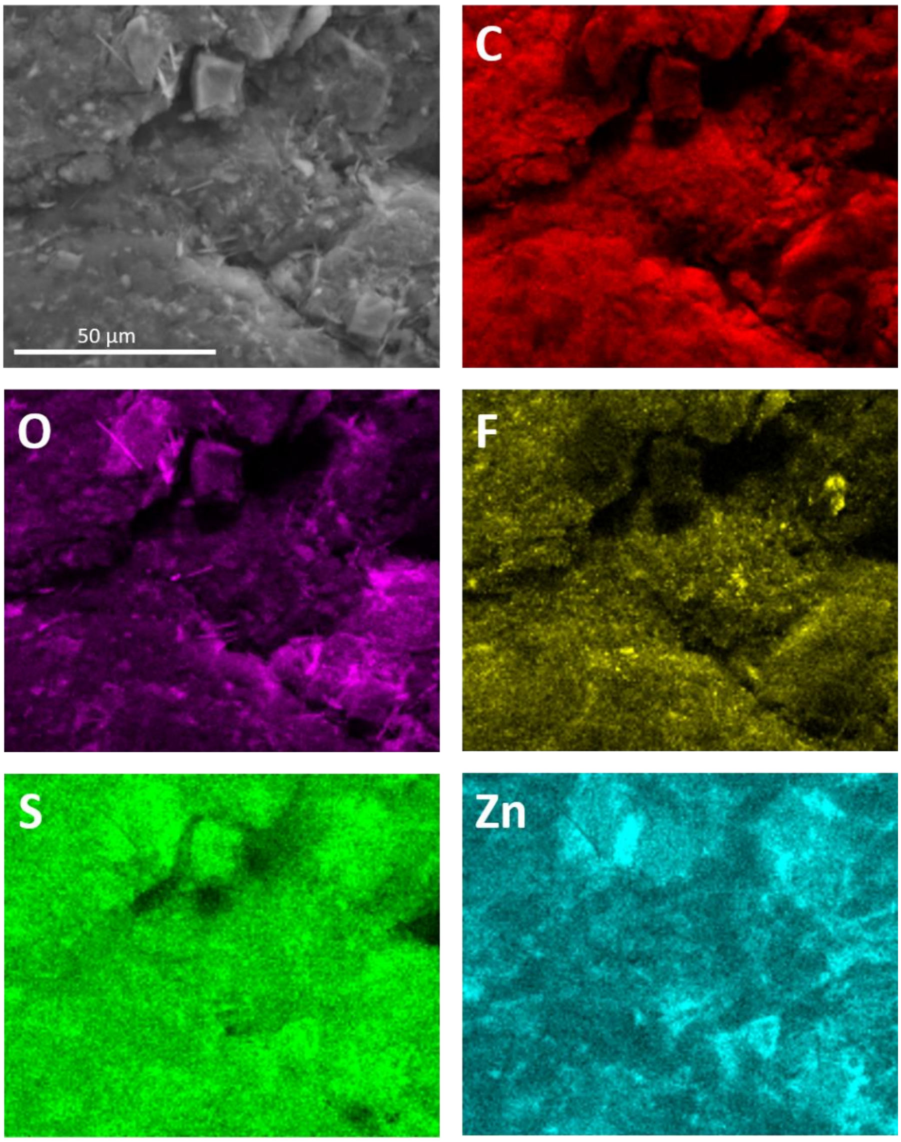

Figure S13: SEM image and EDS Elemental mapping (C, O, F, S and Zn) of OTQC electrode after 5 cycles in Zn battery using 3 M $ZnSO_4$ electrolyte stopped at 0.25 V vs Zn/Zn^{2+}. Average atomic percentages was C: 53%, O: 19%, N: 4%, F: 5%, S: 3%, Zn: 20%*.

Table S1: Comparison of the theoretically calculated values of elemental analysis (orange) and the results of elemental analysis (green). The water content of TQC and OTQC has not been experimentally determined. It is known that quinone compounds are quite hygroscopic containing up to 15 wt. % water content,[4–6] which is matching with the results obtained with elemental analysis.

Elemental analysis	C [wt. %]	H [wt. %]	N [wt. %]	O [wt. %]	wt. % H_2O
TQC (cathechol) theo.	69.50	2.72	21.61	6.17	0
TQC (cathechol) x 3.05 H_2O theo. $C_{30}H_{14}N_8O_2$ x 3.05 H_2O	62.84	3.53	19.54		9.57
TQC (quinone) theo.	69.77	2.34	21.70	6.20	
TQC (quinone) x 3.15 H_2O theo. $C_{30}H_{12}N_8O_2$ x 3.05 H_2O	62.86	3.22	19.55		9.89
OTQC (dimer) theo.	62.51	1.40	19.44	16.65	
OTQC (dimer) x 2.75 H_2O theo. $C_{30}H_{10}N_8O_6$ X 2.75 H_2O	57.38	2.49	17.84		7.88
OTQC (trimer) theo.	64.62	1.55	21.53	12.30	
OTQC (trimer) x 6 H_2O theo. $C_{42}H_{12}N_{12}O_6$ x 6 H_2O	56.76	2.72	18.91		12.15
TQC	63.3	3.1	19.1		
OTQC	56.8	2.3	18.5		

Table S2: Comparison of electrochemical performance of reported small organic cathode materials in Li-organic batteries.

*Note to table: AM – active material, KB – Ketjenblack, PTX – Printex XE2, rGO – reduced graphene oxide, SP – Super P, AB – acetylene black, G – graphene, CB – Vulcan XC-72, PTFE – poly(tetrafluoroethylene), PVDF – poly(vinylidene fluoride), average voltage values have been obtained from ref:[8,9] The cycling time was estimated with the assumption of linear capacity fading using the following equation:

$$t_{estimate} = \frac{C_{max} + Ret * C_{max}}{Curr} * N_{cycles}$$

The calculation for o-IDT, ref:[8]
C_{max}… maximum reversible capacity (273 $mAhg^{-1}$)
Ret… capacity retention after N_{cycles} (82 %)
Curr… current density (50 mAg^{-1})
N_{cycles}… number of cycles (100)
$t_{estimate}$ = 35 days

Cathode material	Electrode composition	Electrolyte	Avg. voltage	Reversible capacity	Capacity retention (cycles)	Capacity retention (estimated time)	Ref.
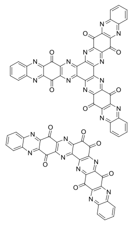 OTQC	AM:PTX:PTFE=60:30:10	1 M LiTFSI in DOL:DME = 1:1 (v/v)	2.63 V	327 $mAhg^{-1}$ @50 mAg^{-1}	93 % after 100 cycles @50 mAg^{-1} 83 % after 400 cycles @50 mAg^{-1}	93 % after 53 days @50 mAg^{-1} 83 % after 194 days @50 mAg^{-1}	This work
		1 M LiPF6 in EC:DMC = 1:1 (v/v)	2.29 V	507 $mAhg^{-1}$ @50 mAg^{-1}	23 % after 60 cycles @50 mAg^{-1}	23 % after 18 days @50 mAg^{-1}	
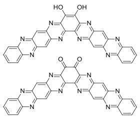 TQC	AM:PTX:PTFE=60:30:10	1 M LiTFSI in DOL:DME = 1:1 (v/v)	2.42 V	223 $mAhg^{-1}$ @50 mAg^{-1}	82 % after 300 cycles @50 mAg^{-1}	82 % after 99 days @50 mAg^{-1}	[3]
		1 M LiPF6 in EC:DMC = 1:1 (v/v)	2.30 V	290 $mAhg^{-1}$ @50 mAg^{-1}	32 % after 50 cycles @50 mAg^{-1}	32 % after 12 days @50 mAg^{-1}	
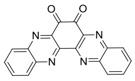 ODQC	AM:PTX:PTFE=60:30:10	1 M LiTFSI in DOL:DME = 1:1 (v/v)	2.58 V	268 $mAhg^{-1}$ @50 mAg^{-1}	19 % after 300 cycles @50 mAg^{-1}	19 % after 29 days @50 mAg^{-1}	[3]
		1 M LiPF6 in EC:DMC = 1:1 (v/v)	2.09 V	484 $mAhg^{-1}$ @50 mAg^{-1}	19 % after 50 cycles @50 mAg^{-1}	19 % after 14 days @50 mAg^{-1}	
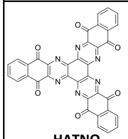 HATNQ	AM:KB:PVDF=60:30:10	1 M LiTFSI in DOL:DME = 1:1 (v/v) + 0.3 wt% $LiNO_3$	2.1 V	225 $mAhg^{-1}$ @200 mAg^{-1}	71 % after 60 cycles @200 mAg^{-1}	71 % after 5 days @200 mAg^{-1}	[7]
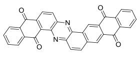 o-IDT	AM:KB:PTFE=60:30:10	1 M LiTFSI in DOL:DME = 1:1 (v/v)	2.45 V	273 $mAhg^{-1}$ @50 mAg^{-1}	82 % after 100 cycles @50 mAg^{-1}	82 % after 41 days @50 mAg^{-1}	[8]
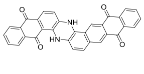 IDT	AM:KB:PTFE=60:30:10	1 M LiTFSI in DOL:DME = 1:1 (v/v)	2.45 V	238 $mAhg^{-1}$ @50 mAg^{-1}	76 % after 100 cycles @50 mAg^{-1}	76 % after 35 days @50 mAg^{-1}	[8]

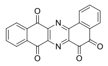 DABTTO	AM:KB:PTFE=60:30:10	1 M LiTFSI in DOL:DME = 1:1 (v/v)	2.53 V	273 $mAhg^{-1}$ @100 mAg^{-1}	43 % after 100 cycles @100 mAg^{-1}	43 % after 16 days @100 mAg^{-1}	[9]
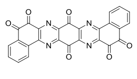 TADBPHO	AM:KB:PTFE=60:30:10	1 M LiTFSI in DOL:DME = 1:1 (v/v)	2.56 V	364 $mAhg^{-1}$ @100 mAg^{-1}	83 % after 100 cycles @100 mAg^{-1}	83 % after 28 days @100 mAg^{-1}	[9]
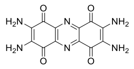 TAPT	AM:SP:PVDF=60:30:10	1 M LiTFSI in DOL:DME = 1:1 (v/v)	2.50 V	353 $mAhg^{-1}$ @100 mAg^{-1}	85 % after 100 cycles @100 mAg^{-1}	85 % after 27 days @100 mAg^{-1}	[10]
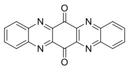 TAPQ	AM:KB:PTFE=60:30:10	1 M LiTFSI in DOL:DME = 1:1 (v/v)	/	260 $mAhg^{-1}$ @100 mAg^{-1}	82 % after 100 cycles @100 mAg^{-1}	82 % after 20 days @100 mAg^{-1}	[2]
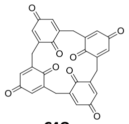 C4Q	AM:KB:PVDF=30:60:10	1 M $LiPF_6$ in EC:DMC = 1:1 (v/v)	2.70 V	427 $mAhg^{-1}$ @45 mAg^{-1}	7 % after 100 cycles @45 mAg^{-1}	7 % after 42 days @45 mAg^{-1}	[11]
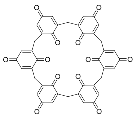 C6Q	AM:KB:PVDF=30:60:10	1 M $LiPF_6$ in EC:DMC = 1:1 (v/v)	2.30 V	423 $mAhg^{-1}$ @45 mAg^{-1}	46 % after 300 cycles @45 mAg^{-1}	46 % after 171 days @45 mAg^{-1}	[12]
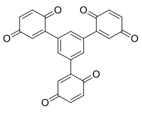 TBQB	AM:G:PVDF=60:30:10	2 M LiTFSI in DOL:DME = 1:1 (v/v)	2.60 V	397 $mAhg^{-1}$ @41 mAg^{-1}	55 % after 100 cycles @45 mAg^{-1}	55 % after 63 days @45 mAg^{-1}	[13]
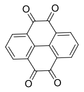 PTO	AM:CB:PVDF=30:50:20	1 M LiPF6 in EC:DMC = 1:1 (v/v)	2.60 V	360 $mAhg^{-1}$ @20 mAg^{-1}	21 % after 50 cycles @20 mAg^{-1}	21 % after 45 days @20 mAg-1	[14]
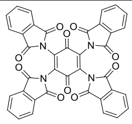 TPB	AM:SP:PVDF=50:40:10	1 M LiTFSI in DOL:DME = 1:1 (v/v)	/	223 $mAhg^{-1}$ @47 mAg^{-1}	91 % after 100 cycles @47 mAg^{-1}	91 % after 38 days @47 mAg^{-1}	[15]
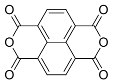 NTCDA	AM:KB:PVDF=60:30:10	1 M LiTFSI in DOL:DME = 1:1 (v/v)	/	212 $mAhg^{-1}$ @50 mAg^{-1}	23 % after 100 cycles @50 mAg^{-1}	23 % after 22 days @50 mAg^{-1}	[16]
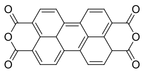 PTCDA	AM:KB:PVDF=60:30:10	1 M LiTFSI in DOL:DME = 1:1 (v/v)	/	154 $mAhg^{-1}$ @50 mAg^{-1}	70 % after 200 cycles @50 mAg^{-1}	70 % after 44 days @50 mAg^{-1}	[16]

Table S3: Comparison of electrochemical performance of reported small organic cathode materials in Zn-organic batteries.

*Note to table: AM – active material, KB – Ketjenblack, PTX – Printex XE2, rGO – reduced graphene oxide, SP – Super P, AB – acetylene black, G – graphene, CB – Vulcan XC-72, PTFE – poly(tetrafluoroethylene), PVDF – poly(vinylidene fluoride), average voltage values have been obtained from ref:[2] For the equation to estimate the cycling time see Table S2.

Cathode Material	Electrode composition	Electrolyte	Avg. Voltage	Reversible capacity	Capacity retention (cycles)	Capacity retention (estimated time)	Reference
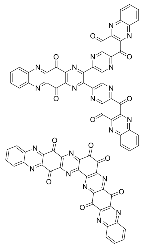 OTQC	AM:PTX:PTFE=60:30:10	3 M $ZnSO_4$	0.86 V	326 $mAhg^{-1}$ @100 mAg^{-1}	60 % after 100 cycles @100 mAg^{-1} 42 % after 400 cycles @100 mAg^{-1}	60 % after 20 days @100 mAg^{-1} 42 % after 60 days @100 mAg^{-1}	This work
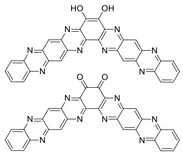 TQC	AM:PTX:PTFE=60:30:10	3 M ZnSO4	0.76 V	301 $mAhg^{-1}$ @50 mAg^{-1}	71 % after 100 cycles @50 mAg^{-1}	71 % after 40 days @50 mAg^{-1}	[3]
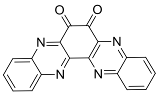 ODQC	AM:PTX:PTFE=60:30:10	3 M ZnSO4	0.56 V	159 $mAhg^{-1}$ @50 mAg^{-1}	89 % after 100 cycles @50 mAg^{-1}	89 % after 9 days @50 mAg^{-1}	[3]
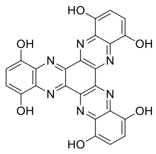 TQD	AM:PTX:PTFE=60:30:10	4 M $ZnSO_4$	0.67 V	503 $mAhg^{-1}$ @100 mAg^{-1}	43 % after 80 cycles @100 mAg^{-1}	43 % after 19 days @100 mAg^{-1}	[4]
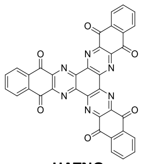 HATNQ	AM:rGO:PVDF=60:35:5	3 M $ZnSO_4$	0.64 V	483 $mAhg^{-1}$ @200 mAg^{-1}	66 % after 100 cycles @500 mAg^{-1}	66 % after 7 days @500 mAg^{-1}	[17]
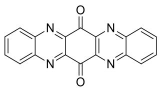 TAPQ	AM:KB:PTFE=60:30:10	1 M $ZnSO_4$	0.64 V	443 $mAhg^{-1}$ @50 mAg^{-1}	50 % after 100 cycles @50 mAg^{-1}	50 % after 55 days @50 mAg^{-1}	[2]
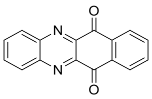 BPD	AM:KB:PVDF=60:30:10	2 M ZnSO4	/	429 $mAhg^{-1}$ @50 mAg^{-1}	74 % after 200 cycles @100 mAg^{-1}	74 % after 62 days @100 mAg^{-1}	[18]

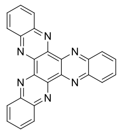 HATN	AM:SP:PVDF=60:35:05	2 M $ZnSO_4$	0.53 V	370 mAhg^{-1} @100 mAg^{-1}	93 % after 5000 cycles @5 Ag^{-1}	74 % after 12 days @100 mAg-1	[19]
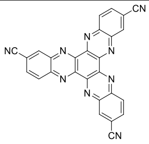 HATN-3CN	AM:KB:PTFE=60:30:10	2 M $ZnSO_4$	0.64 V	320 mAhg^{-1} @50 mAg^{-1}	99.2 % after 150 cycles @300 mAg^{-1}	99.2 % after 13 days @300 mAg^{-1}	[20]
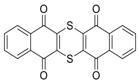 DTT	AM:KB:PTFE=60:30:10	2 M $ZnSO_4$	0.76 V	211 mAhg^{-1} @50 mAg^{-1}	89 % after 150 cycles @100 mAg^{-1}	89 % after 24 days @100 mAg^{-1}	[21]
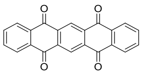 PT	AM:SP:PTFE=55:35:10	2 M $ZnSO_4$	0.6 V	170 mAhg^{-1} @50 mAg^{-1}	95 % after 150 cycles @100 mAg-1	95 % after 20 days @100 mAg-1	[22]
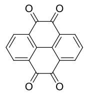 PTO	AM:KB:PTFE=60:30:10	2 M $ZnSO_4$	0.8 V	336 mAhg^{-1} @20 mAg^{-1}	70 % after 1000 cycles @3 Ag^{-1}	70 % after 5 days @3 Ag^{-1}	[23]
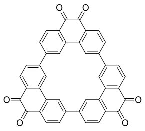 PQ-Δ	AM:AB:PVDF=60:30:10	3 M $Zn(CF_3SO_3)_2$	0.78 V	203 mAhg^{-1} @30 mAg^{-1}	99.9 % after 500 cycles @150 mAg^{-1}	99.9 % after 56 days @150 mAg^{-1}	[24]
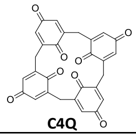 C4Q	AM:SP:PVDF=60:35:05	3 M $Zn(CF_3SO_3)_2$	1.0 V	335 mAhg^{-1} @20 mAg^{-1}	Nafion membrane as separator, 87 % after 1000 cycles @500 mAg^{-1}	87 % after 23 days @500 mAg^{-1}	[25]
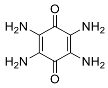 TABQ	AM:KB:PVDF=50:40:10	2 M $ZnSO_4$	/	203 mAhg^{-1} @100 mAg^{-1}	67 % after 100 cycles @160 mAg^{-1}	67 % after 9 days @160 mAg^{-1}	[26]
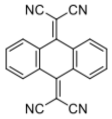 TCNAQ	AM:SP:PTFE=60:30:10	2 M $ZnSO_4$	1.1 V	169 mAhg^{-1} @20 mAg^{-1}	81 % after 1000 cycles @500 mAg^{-1}	81 % after 18 days @500 mAg^{-1}	[27]
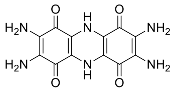 TCBQ	AM:KB:PTFE=80:10:10	1 M $ZnSO_4$	/	369 mAhg^{-1} @20 mAg^{-1}	81 % after 3000 cycles @10 Ag^{-1}	81 % after 4 days @10 Ag^{-1}	[28]
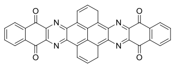 PTONQ	AM:KB:PTFE=50:40:10	2 M $Zn(CF_3SO_3)_2$	/	225 mAhg^{-1} @175 mAg^{-1}	96 % after 200 cycles @175 mAg^{-1}	96 % after 21 days @175 mAg^{-1}	[29]
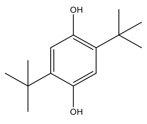 DTBHQ	AM:KB:PTFE=70:25:5	1.25 M $Zn(OAc)_2$	/	110 mAhg^{-1} @50 mAg^{-1}	100 % after 500 cycles @50 mAg^{-1}	100 % after 76 days @50 mAg^{-1}	[30]

References

(1) Li, C.; Kingsbury, R.; Zhou, L.; Shyamsunder, A.; Persson, K. A.; Nazar, L. F. Tuning the Solvation Structure in Aqueous Zinc Batteries to Maximize Zn-Ion Intercalation and Optimize Dendrite-Free Zinc Plating. *ACS Energy Lett.* **2022**, *7* (1), 533–540. https://doi.org/10.1021/acsenergylett.1c02514.

(2) Gao, Y.; Li, G.; Wang, F.; Chu, J.; Yu, P.; Wang, B.; Zhan, H.; Song, Z. A High-Performance Aqueous Rechargeable Zinc Battery Based on Organic Cathode Integrating Quinone and Pyrazine. *Energy Storage Mater.* **2021**, *40* (March), 31–40. https://doi.org/10.1016/j.ensm.2021.05.002.

(3) Menart, S.; Pirnat, K.; Krajnc, A.; Ruiz-Zepeda, F.; Pahovnik, D.; Vélez Santa, J. F.; Dominko, R. Synthesis of Organic Cathode Materials with Pyrazine and Catechol Motifs for Rechargeable Lithium and Zinc Batteries. *J. Power Sources* **2024**, *596*, 234033. https://doi.org/10.1016/j.jpowsour.2023.234033.

(4) Menart, S.; Pirnat, K.; Pahovnik, D.; Dominko, R. Triquinoxalinediol as Organic Cathode Material for Rechargeable Aqueous Zinc-Ion Batteries. *J. Mater. Chem. A* **2023**, *11* (20), 10874–10882. https://doi.org/10.1039/D3TA01400B.

(5) Pirnat, K.; Mali, G.; Gaberscek, M.; Dominko, R. Quinone-Formaldehyde Polymer as an Active Material in Li-Ion Batteries. *J. Power Sources* **2016**, *315*, 169–178. https://doi.org/10.1016/j.jpowsour.2016.03.010.

(6) Pirnat, K.; Casado, N.; Porcarelli, L.; Ballard, N.; Mecerreyes, D. Synthesis of Redox Polymer Nanoparticles Based on Poly(Vinyl Catechols) and Their Electroactivity. *Macromolecules* **2019**, *52* (21), 8155–8166. https://doi.org/10.1021/acs.macromol.9b01405.

(7) Wu, M.; Luu, N. T. H.; Chen, T.; Lyu, H.; Huang, T.; Dai, S.; Sun, X.; Ivanov, A. S.; Lee, J.; Popovs, I.; Kaveevivitchai, W. Supramolecular Self- Assembled Multi-Electron- Acceptor Organic Molecule as High- Performance Cathode Material for Li- Ion Batteries. *Adv. Energy Mater.* **2021**, *11* (31), 2100330. https://doi.org/10.1002/aenm.202100330.

(8) Shi, T.; Li, G.; Han, Y.; Gao, Y.; Wang, F.; Hu, Z.; Cai, T.; Chu, J.; Song, Z. Oxidized Indanthrone as a Cost-Effective and High-Performance Organic Cathode Material for Rechargeable Lithium Batteries. *Energy Storage Mater.* **2022**, *50*, 265–273. https://doi.org/10.1016/j.ensm.2022.05.013.

(9) Chen, Z.; Wang, J.; Cai, T.; Hu, Z.; Chu, J.; Wang, F.; Gan, X.; Song, Z. Constructing Extended π-Conjugated Molecules with o-Quinone Groups as High-Energy Organic Cathode Materials. *ACS Appl. Mater. Interfaces* **2022**, *14* (24), 27994–28003. https://doi.org/10.1021/acsami.2c06252.

(10) Li, Z.; Jia, Q.; Chen, Y.; Fan, K.; Zhang, C.; Zhang, G.; Xu, M.; Mao, M.; Ma, J.; Hu, W.; Wang, C. A Small Molecular Symmetric All- Organic Lithium- Ion Battery. *Angew. Chemie Int. Ed.* **2022**, *61* (33), e2022072. https://doi.org/10.1002/anie.202207221.

(11) Huang, W.; Zheng, S.; Zhang, X.; Zhou, W.; Xiong, W.; Chen, J. Synthesis and Application of Calix[6]Quinone as a High-Capacity Organic Cathode for Plastic

Crystal Electrolyte-Based Lithium-Ion Batteries. *Energy Storage Mater.* **2020**, *26* (September 2019), 465–471. https://doi.org/10.1016/j.ensm.2019.11.020.

(12) Huang, W.; Zhang, X.; Zheng, S.; Zhou, W.; Xie, J.; Yang, Z.; Zhang, Q. Calix[6]Quinone as High-Performance Cathode for Lithium-Ion Battery. *Sci. China Mater.* **2020**, *63* (3), 339–346. https://doi.org/10.1007/s40843-019-1185-2.

(13) Yang, J.; Xiong, P.; Shi, Y.; Sun, P.; Wang, Z.; Chen, Z.; Xu, Y. Rational Molecular Design of Benzoquinone‐ Derived Cathode Materials for High‐ Performance Lithium‐ Ion Batteries. *Adv. Funct. Mater.* **2020**, *30* (15), 1909597. https://doi.org/10.1002/adfm.201909597.

(14) Liang, Y.; Zhang, P.; Chen, J. Function-Oriented Design of Conjugated Carbonyl Compound Electrodes for High Energy Lithium Batteries. *Chem. Sci.* **2013**, *4* (3), 1330–1337. https://doi.org/10.1039/c3sc22093a.

(15) Luo, Z.; Liu, L.; Zhao, Q.; Li, F.; Chen, J. An Insoluble Benzoquinone-Based Organic Cathode for Use in Rechargeable Lithium-Ion Batteries. *Angew. Chemie - Int. Ed.* **2017**, *56* (41), 12561–12565. https://doi.org/10.1002/anie.201706604.

(16) Cai, T.; Han, Y.; Lan, Q.; Wang, F.; Chu, J.; Zhan, H.; Song, Z. Stable Cycling of Small Molecular Organic Electrode Materials Enabled by High Concentration Electrolytes. *Energy Storage Mater.* **2020**, *31*, 318–327. https://doi.org/10.1016/j.ensm.2020.06.032.

(17) Chen, Y.; Li, J.; Zhu, Q.; Fan, K.; Cao, Y.; Zhang, G.; Zhang, C.; Gao, Y.; Zou, J.; Zhai, T.; Wang, C. Two‐ Dimensional Organic Supramolecule via Hydrogen Bonding and π–π Stacking for Ultrahigh Capacity and Long‐ Life Aqueous Zinc–Organic Batteries. *Angew. Chemie Int. Ed.* **2022**, *61* (37), e202116289. https://doi.org/10.1002/anie.202116289.

(18) Shi, Y.; Wang, P.; Gao, H.; Jin, W.; Chen, Y.; Huang, Y.; Wu, T.-R.; Wu, D.-Y.; Xu, J.; Cao, J. π-Conjugated N-Heterocyclic Compound with Redox-Active Quinone and Pyrazine Moieties as a High-Capacity Organic Cathode for Aqueous Zinc-Ion Batteries. *Chem. Eng. J.* **2023**, *461*, 141850. https://doi.org/10.1016/j.cej.2023.141850.

(19) Tie, Z.; Liu, L.; Deng, S.; Zhao, D.; Niu, Z. Proton Insertion Chemistry of a Zinc–Organic Battery. *Angew. Chemie Int. Ed.* **2020**, *59* (12), 4920–4924. https://doi.org/10.1002/anie.201916529.

(20) Ye, Z.; Xie, S.; Cao, Z.; Wang, L.; Xu, D.; Zhang, H.; Matz, J.; Dong, P.; Fang, H.; Shen, J.; Ye, M. High-Rate Aqueous Zinc-Organic Battery Achieved by Lowering HOMO/LUMO of Organic Cathode. *Energy Storage Mater.* **2021**, *37*, 378–386. https://doi.org/10.1016/j.ensm.2021.02.022.

(21) Wang, Y.; Wang, C.; Ni, Z.; Gu, Y.; Wang, B.; Guo, Z.; Wang, Z.; Bin, D.; Ma, J.; Wang, Y. Binding Zinc Ions by Carboxyl Groups from Adjacent Molecules toward Long‐ Life Aqueous Zinc–Organic Batteries. *Adv. Mater.* **2020**, *32* (16), 2000338. https://doi.org/10.1002/adma.202000338.

(22) Mirle, C.; Medabalmi, V.; Ramanujam, K. Electrode and Conductive Additive Compatibility Yielding Excellent Rate Capability and Long Cycle Life for Sustainable Organic Aqueous Zn-Ion Batteries. *ACS Appl. Energy Mater.* **2021**, *4* (2), 1218–1227. https://doi.org/10.1021/acsaem.0c02511.

(23) Guo, Z.; Ma, Y.; Dong, X.; Huang, J.; Wang, Y.; Xia, Y. An Environmentally Friendly and Flexible Aqueous Zinc Battery Using an Organic Cathode. *Angew. Chemie - Int. Ed.* **2018**, *57* (36), 11737–11741. https://doi.org/10.1002/anie.201807121.

(24) Nam, K. W.; Kim, H.; Beldjoudi, Y.; Kwon, T. W.; Kim, D. J.; Stoddart, J. F. Redox-Active Phenanthrenequinone Triangles in Aqueous Rechargeable Zinc Batteries. *J. Am. Chem. Soc.* **2020**, *142* (5), 2541–2548. https://doi.org/10.1021/jacs.9b12436.

(25) Zhao, Q.; Huang, W.; Luo, Z.; Liu, L.; Lu, Y.; Li, Y.; Li, L.; Hu, J.; Ma, H.; Chen, J. High-Capacity Aqueous Zinc Batteries Using Sustainable Quinone Electrodes. *Sci. Adv.* **2018**, *4* (3), eaao1761. https://doi.org/10.1126/sciadv.aao1761.

(26) Lin, Z.; Shi, H. Y.; Lin, L.; Yang, X.; Wu, W.; Sun, X. A High Capacity Small Molecule Quinone Cathode for Rechargeable Aqueous Zinc-Organic Batteries. *Nat. Commun.* **2021**, *12*, 4424. https://doi.org/10.1038/s41467-021-24701-9.

(27) Wang, Q.; Xu, X.; Yang, G.; Liu, Y.; Yao, X. An Organic Cathode with Tailored Working Potential for Aqueous Zn-Ion Batteries. *Chem. Commun.* **2020**, *56* (79), 11859–11862. https://doi.org/10.1039/D0CC05344A.

(28) Lin, L.; Lin, Z.; Zhu, J.; Wang, K.; Wu, W.; Qiu, T.; Sun, X. A Semi-Conductive Organic Cathode Material Enabled by Extended Conjugation for Rechargeable Aqueous Zinc Batteries. *Energy Environ. Sci.* **2023**, *16* (1), 89–96. https://doi.org/10.1039/D2EE02961H.

(29) Sun, T.; Yi, Z.; Zhang, W.; Nian, Q.; Fan, H. J.; Tao, Z. Dynamic Balance of Partial Charge for Small Organic Compound in Aqueous Zinc- Organic Battery. *Adv. Funct. Mater.* **2023**, *33* (47), 2306675. https://doi.org/10.1002/adfm.202306675.

(30) Singh, A.; Grenz, D.; Pellegrin, Y.; Odobel, F.; Poizot, P.; Gaubicher, J. Challenging Metal-Ion Rocking-Chair and Zinc-Ion Mechanisms in Mild Acidic to Neutral Aqueous Electrolytes. *Electrochem. commun.* **2023**, *154*, 107559. https://doi.org/10.1016/j.elecom.2023.107559.
